# New hope for tumor immunotherapy: the macrophage-related “do not eat me” signaling pathway

**DOI:** 10.3389/fphar.2023.1228962

**Published:** 2023-07-06

**Authors:** Han Deng, Guan Wang, Shengyan Zhao, Yiran Tao, Zhixiong Zhang, Jinliang Yang, Yi Lei

**Affiliations:** ^1^ General Practice Ward/International Medical Center, General Practice Medical Center, State Key Laboratory of Biotherapy and Cancer Center, West China Hospital, West China School of Nursing, Sichuan University, Chengdu, Sichuan, China; ^2^ West China-California Research Center for Predictive Intervention Medicine, West China Hospital, Sichuan University, Chengdu, Sichuan, China

**Keywords:** “do not eat me” signaling pathway, CD47, CD24, antibody, small molecule drugs

## Abstract

The “do not eat me” signaling pathway is extremely active in tumor cells, providing a means for these cells to elude macrophage phagocytosis and escape immune surveillance. Representative markers of this pathway, such as CD47 and CD24, are highly expressed in numerous tumors. The interaction of SIRPα with CD47 reduces the accumulation of non-myosin ⅡA on the cell membrane. The combination of CD24 and Siglec10 ultimately leads to the recruitment of SHP-1 or SHP-2 to reduce signal transduction. Both of them weaken the ability of macrophages to engulf tumor cells. Blocking the mutual recognition between CD47-SIRPα or CD24-Siglec10 using large molecular proteins or small molecular drugs represents a promising avenue for tumor immunotherapy. Doing so can inhibit signal transduction and enhance macrophage clearance rates of cancer cells. In this paper, we summarize the characteristics of the drugs that affect the “do not eat me” signaling pathway via classical large molecular proteins and small molecule drugs, which target the CD47-SIRPα and CD24-Siglec10 signaling pathways, which target the CD47-SIRPα and CD24-Siglec10 signaling pathways. We expect it will offer insight into the development of new drugs centered on blocking the “do not eat me” signaling pathway.

## 1 Introduction

**FIGURE 1:**
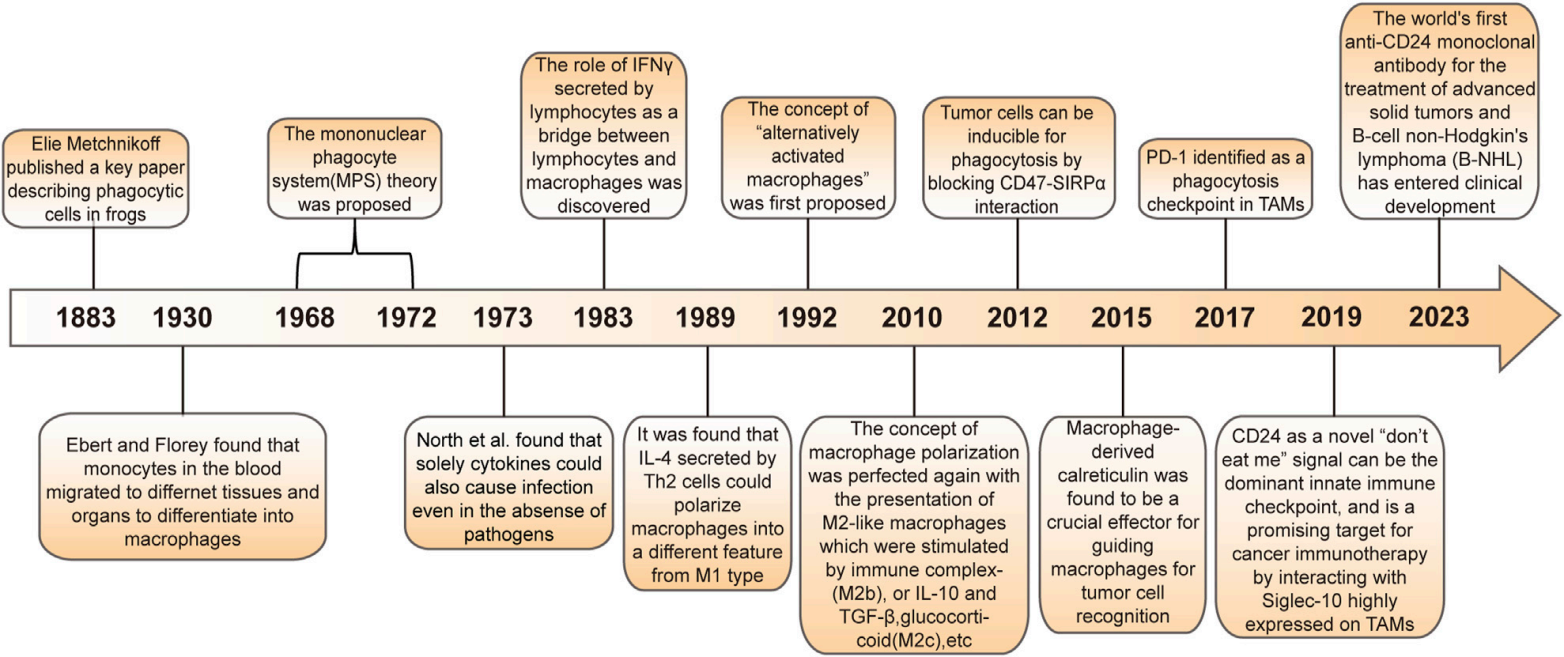
History of macrophages in cancer.

Macrophages are a type of phagocyte that were originally discovered by the Ukrainian biologist, Elie Metchnikoff in the 19th century (ElliottKosterMurphy, 2017; KlocKubiak, 2021). In recent years, they have been increasingly recognized as cells involved in tumor immunotherapy (Figure 1). Depending on their phenotype and function, macrophages differentiate into M1 type (which kill bacteria and viruses, activate immunity, and have an anti-tumor effect) and M2 type (which kill parasites, participate in angiogenesis, wound healing, and have an immunosuppressive role) (Orecchioni et al., 2019; Yunna et al., 2020). Macrophages affect other immune cells by secreting various factors. For example, macrophages can secrete vascular endothelial growth factor (VEGF) and epidermal growth factor (EGF) that promote tumor growth (Harmey et al., 1998; Lu et al., 2015; Gratchev, 2017). Nitric oxide can inhibit tumor growth, and macrophages can induce the production of nitric oxide synthase to enhance the anti-tumor effect of macrophages (Nath and Kashfi, 2020). Macrophage-based cancer therapies reduce M2 macrophages and increase M1 macrophages, thereby suppressing tumor growth.

**FIGURE 2:**
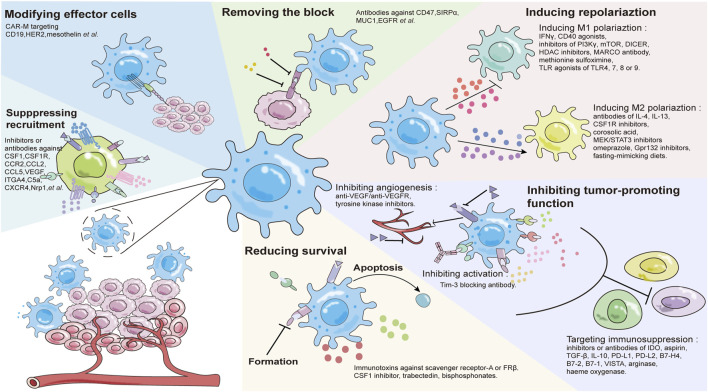
Tumor therapeutic strategies targeting macrophages.

Macrophages play a pivotal role in the formation of key features such as tumor blood vessel production, metastasis and tumor microenvironment regulation. In tumor immunotherapy, there are fewer invasive T cells, leading to differences in the efficacy of T cell-based anti-tumor therapy. However, in the specific environment of tumor tissue, macrophages represent 30%–50% of infiltrating immune cells which makes macrophage-based immunotherapy is more advantageous. Tumor associated macrophages (TAMs) promote tumor growth and invasion, most of which are anti-inflammatory M2 subtypes. In the early stage of tumor development, M1 macrophages in tumor tissues express high levels of pro-inflammatory cytokines (such as IFN-γ and TNF-α), directly kill cancer cells, and have the ability to activate the adaptive immune system, which makes M1 macrophages very important in tumor therapy (Nie et al., 2020). However, in the process of tumor development, tumor tissues will polarize M1 macrophages into M2 immunosuppressive macrophages and participate in the process of tumor metastasis (Feng et al., 2019). Therefore, macrophages have become one of the important targets of tumor therapy. Macrophage-based therapy is likely to achieve significant success in the clinical practice, because peripheral blood mononuclear cells (PBMCs) (the primary source of infiltrating macrophages in tumors) are convenient and easy to collect. Current strategies that target macrophages for effective tumor immunotherapy include: 1) inhibition of macrophage recruitment; 2) reduction of macrophage survival rates; 3) modification of effector cells; 4) relieving the blocking of macrophages; 5) induction of phenotypic polarization; 6) inhibition of tumor-promoting function (Figure 2) (Komohara et al., 2015; Jiang et al., 2016; Lee et al., 2016; Yang et al., 2019; Di Conza et al., 2021; Duan and Luo, 2021; Li et al., 2021; Santoni et al., 2021; Sloas et al., 2021; Tian et al., 2021).

“Do not eat me” signaling proteins is one that is present in normal cells but expressed at higher levels in tumor cells. The activation of this signal can prevent the body’s immune system from detecting and destroying tumor cells (Dolgin, 2018). Weissman’s team found that almost all cancer cells had high levels of CD47, and the large amount of CD47 expression interacts with macrophages’ SIRPα, which the tumor clearance ability of macrophages (Jaiswal et al., 2009; Majeti et al., 2009). Drugs targeting CD47 can enhance macrophage phagocytosis in the special environment of tumor tissue and promote macrophage recruitment to tumor by directly blocking the binding of SIRPα to CD47 (Tseng et al., 2013). In a new study, Weissman and his colleagues identified a novel “do not eat me” signaling protein CD24. CD24 transmits inhibitory signals to reduce macrophage phagocytosis. The team’s findings suggest that antibodies that block CD24 and Siglec10 interactions can enhance macrophages’ effectiveness in killing tumor cells in refractory ovarian cancer, which is common in women, and triple-negative breast cancer, which is difficult to immunotherapy. By contrast, CD24-blocking antibodies did not have a significant therapeutic effect on CD24-deficient tumor cells (Barkal et al., 2019). Additionally, Weissman’s team found that a protein called Major Histocompatibility Complex Class I (MHC-1) can weaken the binding interaction between macrophages and cancerous cells in tumor tissue. They found that human tumors with high surface MHC-1 expression were less sensitive to CD47-related drugs (such as CD47 antibodies) compared to tumors with low surface MHC-1 expression (Barkal et al., 2018). Unfortunately, the mechanism by which MHC-1 interacts with macrophages is still unclear and will not be discussed here (Zhao et al., 2019).

At present, the research and development of drugs that target the “do not eat me” signaling pathway is a hot topic in the new generation of tumor immunotherapy, with drugs that focus on the CD47-SIRPα axis show rapid progress in clinical development (Supplementary Table S1). Magrolimab (Sikic et al., 2019), a monoclonal antibody targeting CD47, and RRx-001 (Oronsky et al., 2021a), a small-molecule inhibitor, have made the most rapid progress in clinical trials. Magrolimab is an immunotherapy agent for myelodysplastic syndromes (MDS) and acute myeloid leukemia (AML) (NCT04778397), and phase II trials of Magrolimab for non-Hodgkin’s lymphoma (NHL) are currently underway (NCT02953509) (Advani et al., 2018). RRx-001 is a small molecule interfering compound and is in Phase III clinical development. It can affect the normal transduction of CD47-SIRPα pathway. It has exhibited greater effectiveness when combined with platinum-based chemotherapy drugs in small cell lung cancer (SCLC) (NCT03699956). In addition, RRx-001 is also in phase II trials in colorectal cancer (NCT02096354), non-small cell lung cancer (NSCLC) (NCT02489903), neuroendocrine carcinoma (NCT02489903), and ovarian cancer (NCT02489903) (Lee et al., 2021) and is currently being studied for potential use in treating glioma (Oronsky et al., 2021b).

Macrophages provide numerous options for tumor immunotherapy, and their ability should not be underestimated. However, there is currently no definitive review of drugs related to the “do not eat me” signaling pathway. Thus, this article aims to review the progress of research on drugs related to the “do not eat me” signaling pathway with an emphasis on small molecule drugs related to CD47-SIRPα. Our goal is to provide guidance for future research on drugs related to the “do not eat me” signaling pathway.

## 2 “Do not eat me” signaling pathway

The “do not eat me” signaling proteins are typically cell membrane proteins that serve to prevent macrophages from destroying them. In recent years, researchers have discovered that tumor cells able to evade attack from immune cells by overexpressing the “do not eat me” signaling protein. When the “do not eat me” signaling pathway is blocked, macrophages are able to effectively kill tumor cells. Therefore, the “do not eat me” signaling protein has become a novel target for immunotherapy, which differs from T cell immune checkpoint.

Currently, the CD47-SIRPα axis and CD24-Siglec10 axis are under detailed study in the “do not eat me” signaling pathways. CD47 binds to SIRPα, resulting in the phosphorylation of their immunoreceptor tyrosine inhibitory motif (ITIM) and subsequent recruitment SHP-1 protein, resulting in a series of cascades that inhibit macrophage phagocytosis (Matlung et al., 2017). In 2019, the CD24-Siglec10 axis becomes the second identified “Do not eat me” signaling pathway after CD47-SIRPα axis. Through overexpression of CD24, tumor cells interact with Siglec10, which inhibits the activity of macrophages and achieves immune escape (Altevogt et al., 2020).

### 2.1 CD47-SIRPα signaling pathway

CD47 is a five-transmembrane receptor belonging to the immunoglobulin superfamily, also known as integrin-associated protein (IAP). It is present in various tissue cells and was initially observed to bind integrin αvβ3 (Brown et al., 1990). CD47 performs distinct functions in different tissues (Lindberg et al., 1994; Moralez et al., 2005), such as inhibiting dendritic cells, regulating integrin secretion, participating in neutrophil migration, and platelet activation. CD47’s N-terminal extracellular domain features a complex IgV-like structure that interacts well with the N-terminal IgV-like structure of signal regulatory protein α (SIRPα) (Hatherley et al., 2008). Additionally, five domains of CD47 are anchored to the cell membrane, while its intracellular domain is very short. CD47 binds to the SIRPα receptor to prevent immature dendritic cells from maturing and inhibits cytokine production by mature dendritic cells. Aside from binding to SIRPα, CD47 can also bind to SIRPγ of T cells to stimulate and activate T cells synergistically, and interaction between CD47 and SIRPγ on superantigen dependent T cells can enhance T cell proliferation (Piccio et al., 2005).

SIRPα is a class of IgSF transmembrane proteins that features a SHPS-1 domain containing the SH2 domain. The extracellular domain links to a domain containing ITIM through a transmembrane region (Zhang et al., 2020). SIRPα contains two tyrosine phosphorylation sites that bind to protein tyrosine phosphatases, such as SHP-1 and SHP-2, to activate them (Murata et al., 2014). Despite its high degree of extracellular homology with SIRPα, SIRPγ is incapable of conducting signaling due to the short intracellular domain of SIRPγ. This is the main reason why SIRPγ cannot effectively recruit signaling proteins and lacks charged residues to participate in the association with DAP12 or other adaptor proteins (Voets et al., 2019).

**FIGURE 3:**
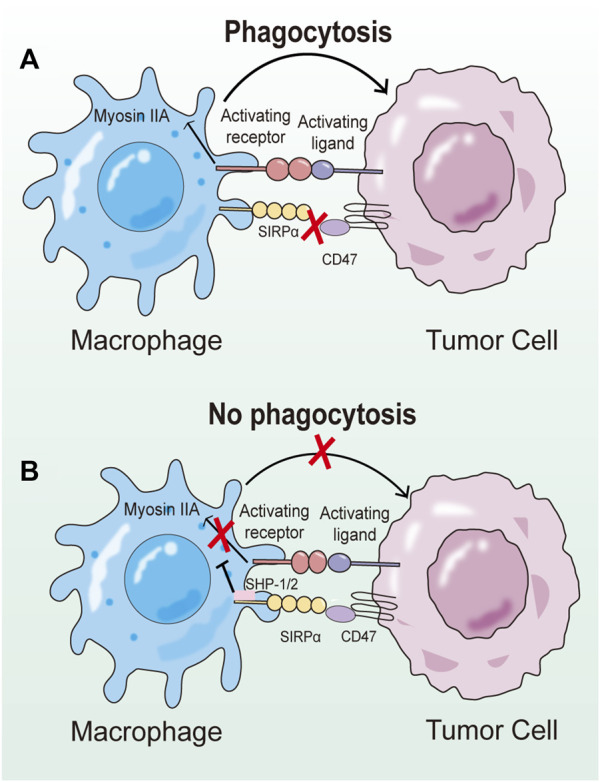
Schematic diagram of the regulatory mechanism of CD47-SIRPα axis on phagocytosis. **(A)** Upon binding to CD47, the ITIM of SIRPα is phosphorylated, and then SHP-1 and SHP-2 are recruited and activated at relevant sites. SHP-1 and SHP-2 reduce the ability of prephagocytosis receptors to trigger phagocytosis, but the exact mechanism remains unclear. **(B)** When CD47-SIRPα signaling pathway is blocked, prophagocytic receptors can induce activation signals to induce phagocytosis of target cells by macrophages.

The CD47-SIRPα axis is the first macrophage-related checkpoint identified for tumor immunotherapy in the 21st century. The X-ray crystal structure highlights that the primary interaction occurs between the FG-loop formed by β-folding in CD47 and the large pocket formed by BC, CD, DE, and FG loops in SIRPα. They form a complex contact surface, where the force is derived from charge complementarities (Hatherley et al., 2008). SHP1 and SHP2 of SIRPα are recruited and activated by phosphorylated tyrosine residues. Ultimately, this transduction process leads to the dephosphorylation of myosin IIA, inhibiting cytoskeletal rearrangement and preventing macrophage phagocytosis. The effect of the CD47-SIRPα axis on the proliferation and survival of tumor cells is indirect. Even in the absence of multiple lymphocytes, blocking this signaling pathway can reduce the number of tumor cells co-incubated with macrophages. This suggests that tumor cells expressing CD47 can avoid macrophage phagocytosis, allowing them to escape being cleared by macrophages (Figure 3) (Lian et al., 2019).

### 2.2 CD24-Siglec10 signaling pathway

CD24 is a highly glycosylated protein with excellent thermal stability, and its solubility in organic solvents and thermal stability is due to its membrane glycoprotein lipid-like structure. CD24 is attached to the plasma membrane by anchoring it with glycosylphosphatidylinositol. The segment encoding CD24 in the human genome was localized to chromosome 6q21, and CD24 pseudogenes without introns have been identified on autosomes 1p36, 15q212q22, and sex chromosome Yq11. Human CD24 has more serine and threonine residues than murine CD24, which gives it a more characteristic mucin-like structure. Binding of CD24’s glycogroup to the cell membrane’s lectin-like ligand can trigger a differentiation signal, while the release of a second messenger from the GPI-anchor transduces differentiation signals. Furthermore, CD24 also regulates B cell activation, decreasing the number of antibody-forming B cells (Suzuki et al., 2001).

Siglecs (sialic acid-binding immunoglobulin-like lectin) are a class of type I transmembrane proteins that serve as cell surface receptors. Structurally, most Siglecs have a typical N-terminal IgV-like domain. One or more ITIMs are present in most Siglecs, which play a role in their negative regulatory signaling function (Munday et al., 2001). Siglec10 is expressed in human leukocytes, and its ligands include CD24, CD52, and vascular adhesion protein-1 (VAP-1). The present study reveals that Siglec10 is an inhibitory receptor in the innate immune system, and its inhibitory function is achieved through the cytosolic domain of Siglec10, which is related to tyrosine phosphatase (Zhang et al., 2015). Upon tyrosine phosphorylation, ITIM recruits SH2 family phosphatases such as SHP-1 and PTPN6. HSP70, HSP90 and HMGB1 belong to danger associated molecular patterns (DAMPs), whereas CD24 and human Siglec10-mediated signaling pathway parameters have selective inhibition of immune response to DAMPs (Chen et al., 2009).

**FIGURE 4:**
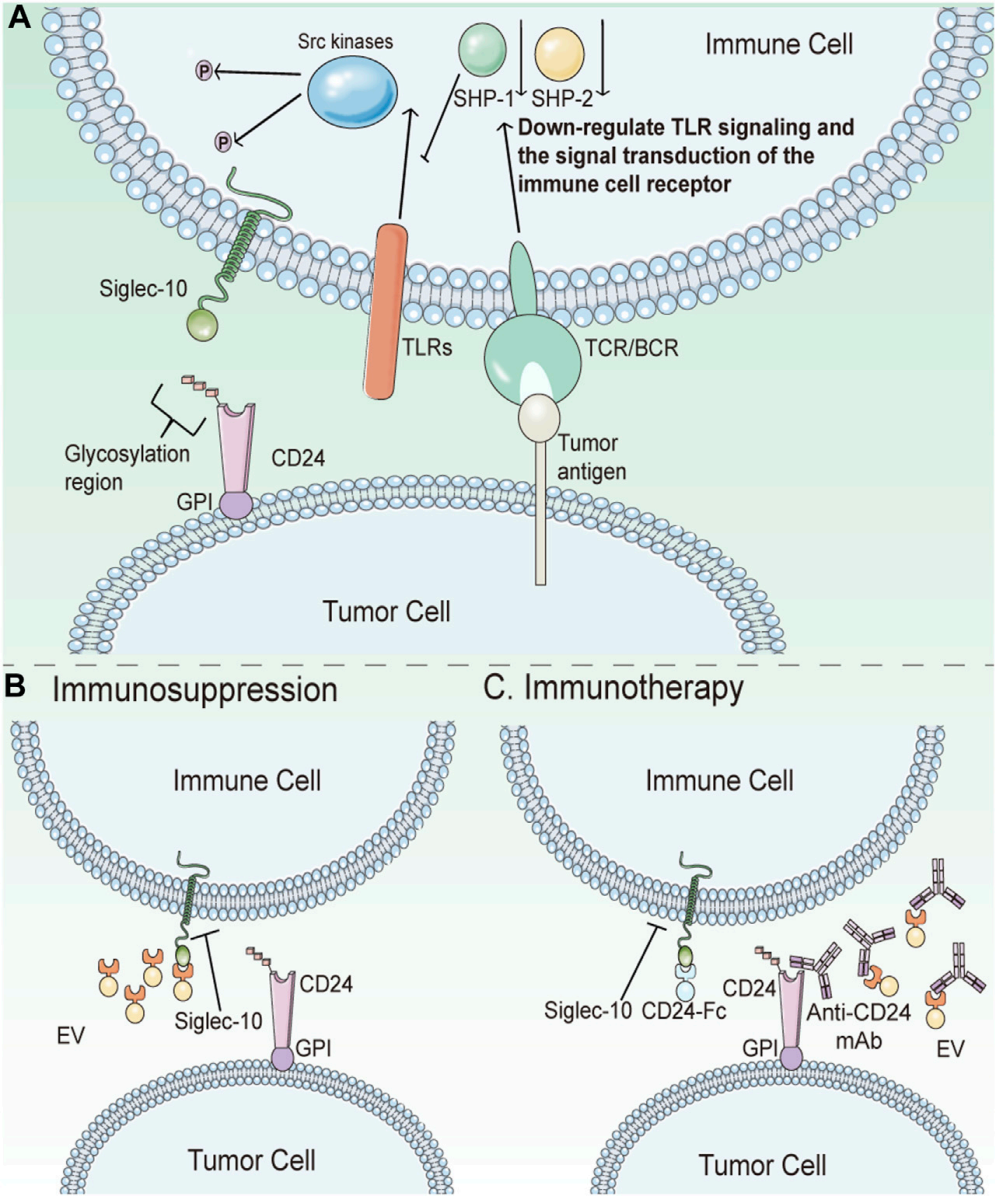
Schematic diagram of CD24 in tumor immunotherapy. **(A)** CD24 helps cancer cells escape phagocytosis by interacting with Siglec10. Src family kinases are induced by ITIM or ITIM-like motifs when Siglec10’s IgV domain binds to sialic acid fragments located in the CD24 extracellular domain. After that, phosphorylated ITIM tyrosine in the cytoplasm recruits tyrosine phosphatase, thereby reducing signal transduction of the CD24-Siglec10 axis. This process will inhibit the immune activity of macrophages, reduce the phagocytosis of macrophages, and weaken the immune surveillance on tumor cells. **(B)** Tumor-derived extracellular vesicles (EV) are not phagocytic due to the expression of CD24 on them. **(C)** Anti-CD24 antibodies can eliminate the inhibition of CD24 on tumor cells or EV surface to macrophages and restore the clearance ability of macrophages. As an immunotherapy, CD24-Fc can trigger immunosuppression of Siglec-10 expressing immune cells, thereby protecting autoimmunity. In cancer, it is deleterious. However, CD24-Fc can block signal transduction between CD24-Sigle10 by competing as an inhibitor of CD24.

CD24 and its ligands cannot be disregarded as participants in the formation of key features of cancer. Inhibiting their interaction may be one of the anticancer therapeutic approaches. CD24 is abnormally overexpressed in many tumors compared to normal tissues, and Siglec10 expression is also higher in tumor-associated macrophages. Src family kinases are activated by ITIM or ITIM-like motifs when the IgV domain of Siglec10 binds to sialic acid fragments located in the CD24 extracellular domain. Following this, phosphorylated ITIM tyrosine in the cytoplasm recruits tyrosine phosphatase, which reduces signal transduction of the CD24-Siglec10 axis. This process inhibits the immune activity of macrophages, reduces macrophage phagocytosis, and weaken the immune surveillance on tumor cells (Figure 4) (Crocker et al., 2007). Knockdown of CD24 or Siglec10, as well as using CD24 monoclonal antibodies to block the interaction of CD24 with Siglec10, significantly enhances macrophage phagocytosis of all CD24-expressing human tumor cells. Weissman et al. discovered that CD24-Siglec10 inhibits macrophage phagocytosis to promote immune evasion in particular CD24-expressing cancer cells (Altevogt et al., 2020). Furthermore, the study has shown that the interaction of CD24 and Siglec10 can produce immunosuppressive signals, resulting in natural killer cells (NK) dysfunction, allowing hepatocellular carcinoma (HCC) cells to avoid NK cytotoxicity of tumor cells (Chen et al., 2009).

In conclusion, the “do not eat me” signaling pathway shares two common features. Firstly, a target of the pathway is also present in normal tissue cells, but is abnormally overexpressed in tumor cells. Secondly, another target in the pathway, such as SIRPα and Siglec10, is a membrane receptor expressed by macrophages, and the activation of this target inhibits the transduction of downstream signals. Abnormally high expression of “do not eat me” signaling proteins, binding to macrophage surface related receptors, like SIRPα and Siglec10, can impair relevant signaling in macrophages, leading to reduced macrophage clearance and ineffective immune surveillance against tumor cells. The specific blockade of the “do not eat me” signaling pathway can enhance the killing effect of macrophages on tumor cells. Based on this principle, blocking antibodies and small molecule inhibitors against CD47-SIRPα and CD24-Siglec10 signaling pathways have been developed.

## 3 Development of drugs related to CD47-SIRPα signaling pathway

### 3.1 Discovery of CD47-SIRPα signaling pathway

**FIGURE 5:**
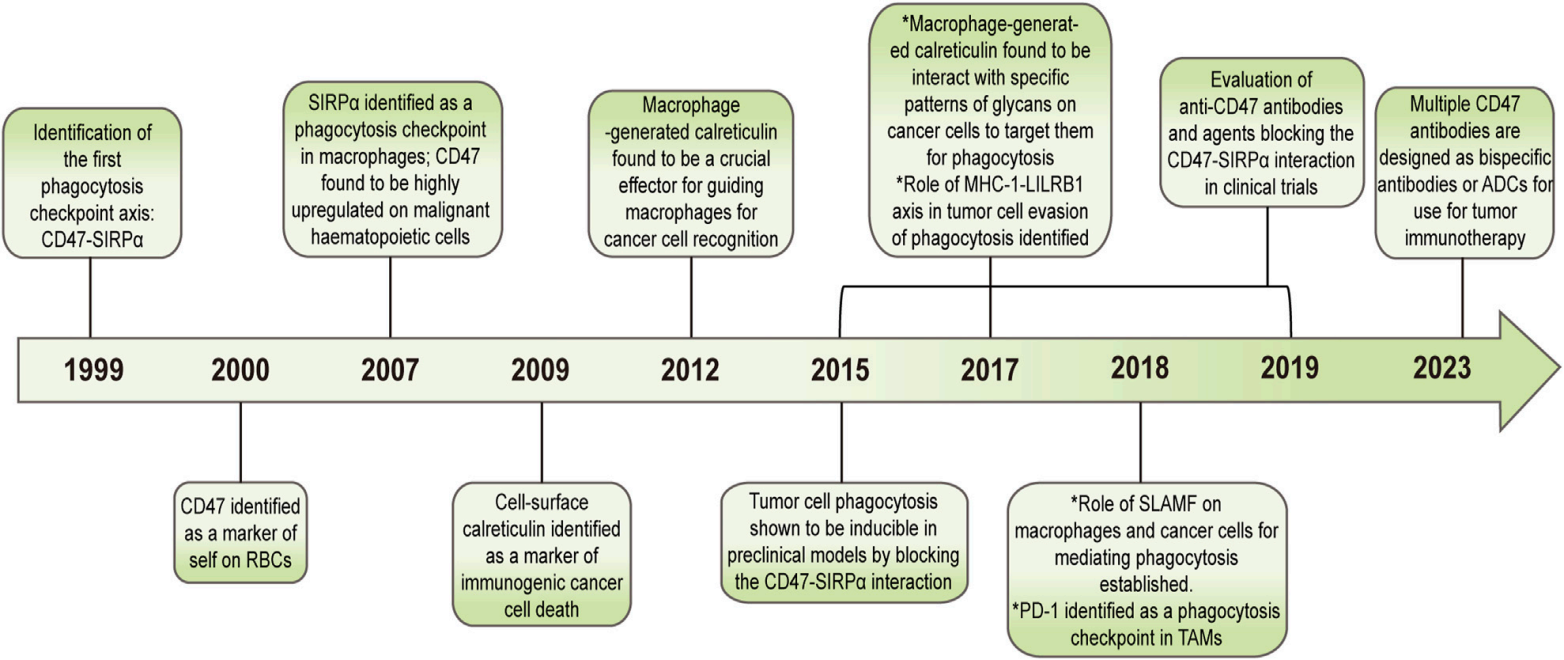
Drug development that blocks CD47 as the phagocytosis checkpoint in cancer.

In the 1990 s, researchers discovered the first phagocytosis checkpoint in macrophages and recognized CD47 and SIRPα as proteins associated with this regulatory signaling pathway, with CD47 being subsequently identified as a “self-marker” on red blood cells (RBCs). In 2009, researchers found abnormal expression of CD47 in both malignant and non-hematopoietic cells. Tumor cells escape phagocytosis through high expression of CD47, which has been demonstrated *in vitro* and *in vivo*. Interference with CD47 inhibition of macrophages using antibodies has been found to slow tumor implantation and growth in preclinical models (Majeti et al., 2009; Willingham et al., 2012). As such, CD47 has been gradually used in cancer treatment, leading to the development of relevant blocking drugs targeting CD47 (Figure 5).

Cell surface calreticulin (CRT) is a key protein marker that identifies the death of immunogenic cancer cells. In 2015, researchers discovered that macrophage-produced CRT is a key effector that directs macrophages to recognize cancer cells. Additionally, specific patterns of glycans on tumor cells have been identified as interacting with macrophage CRTs, with macrophages targeting these specific patterns of glycans for phagocytosis (Feng et al., 2018). Signaling lymphocyte activation molecular family 7 (SLAMF7) has been identified as a non-negligible component of CD47-mediated macrophage clearance of hematopoietic tumor cells (Chen et al., 2017). Furthermore, in 2017 and 2018, PD-1 and leukocyte immunoglobulin-like receptor B (LILRB1) were identified as additional macrophage modulators through their interactions with PD-L1 and MHC-I to regulate macrophage phagocytotic effect on tumor cells (Feng et al., 2019).

### 3.2 CD47-SIRPα signaling pathway related antibodies and fusion proteins

**FIGURE 6:**
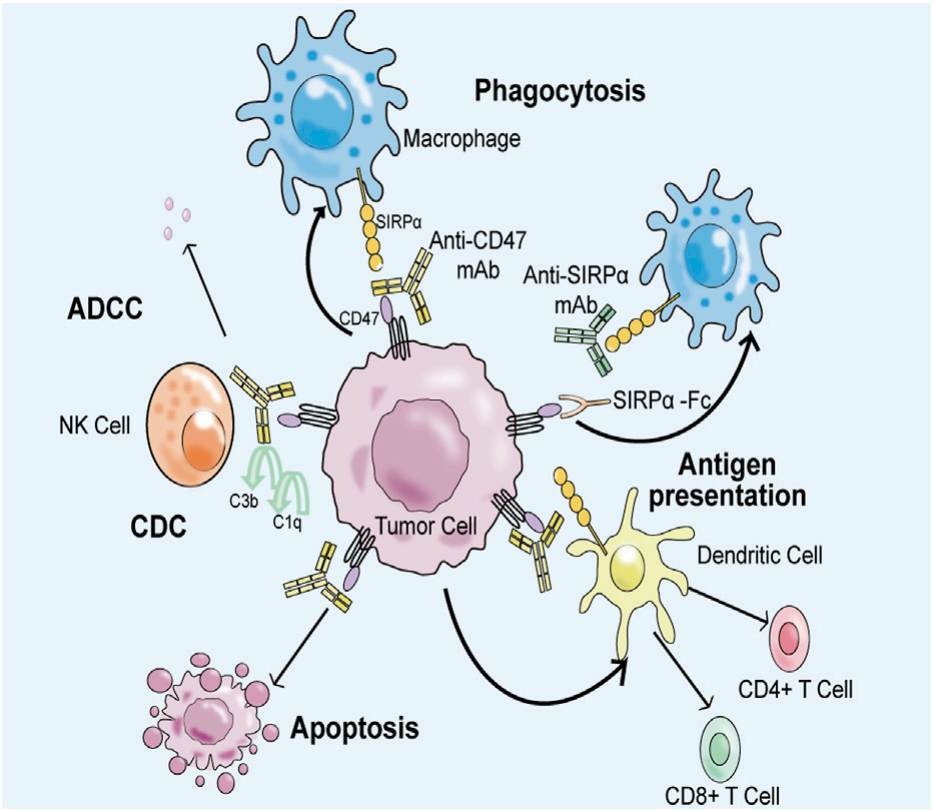
Anti-CD47-SIRPα axis antibody can block CD47-SIRPα signal transduction, restore the immune surveillance ability and immune clearance ability of macrophages to tumor cells. Firstly, negative regulatory signal transduction involving CD47 and SIRPα is inhibited, which improves the clearance efficiency of macrophages on tumor cells. Second, anti-CD47 antibodies induce an adaptive immune response to kill tumor cells. Thirdly, anti-CD47 antibodies bind to natural killer cells via the Fc portion, producing antibody-dependent cytotoxic effects (ADCC) and complement-dependent cytotoxic effects (CDC) to eliminate tumor cells. Finally, CD47-related antibodies promote the apoptosis process of tumor cells.

Tumor cells can effectively evade immune surveillance and clearance mechanisms by overexpressing CD47. However, the use of biomacromolecular drugs that specifically target CD47 or SIRPα has shown to be effective in activating immune responses and combating tumors. For instance, anti-CD47 antibody or CD47 fusion protein can block the signal transduction in this pathway, thus enhancing the recruitment of macrophages to tumor tissues and leading to increased the immune clearance ability of macrophages to tumor cells. Furthermore, antibodies that target CD47 can stimulate adaptive immune responses and activate innate immune responses through antibody-dependent cytotoxicity. Inhibition of the CD47-SIRPα signaling pathway has also been shown to reduce the proliferation of tumor cells and accelerate the apoptosis process (Figure 6) (Kim et al., 2008; Tseng et al., 2013; Weiskopf et al., 2016). As such, CD47 represents a promising immune drug target in cancer immunotherapy. This article briefly introduces three leading anti-CD47-SIRPα antibodies: Hu5F9-G4, Evorpacept, and Lemzoparlimab.

**FIGURE 7:**
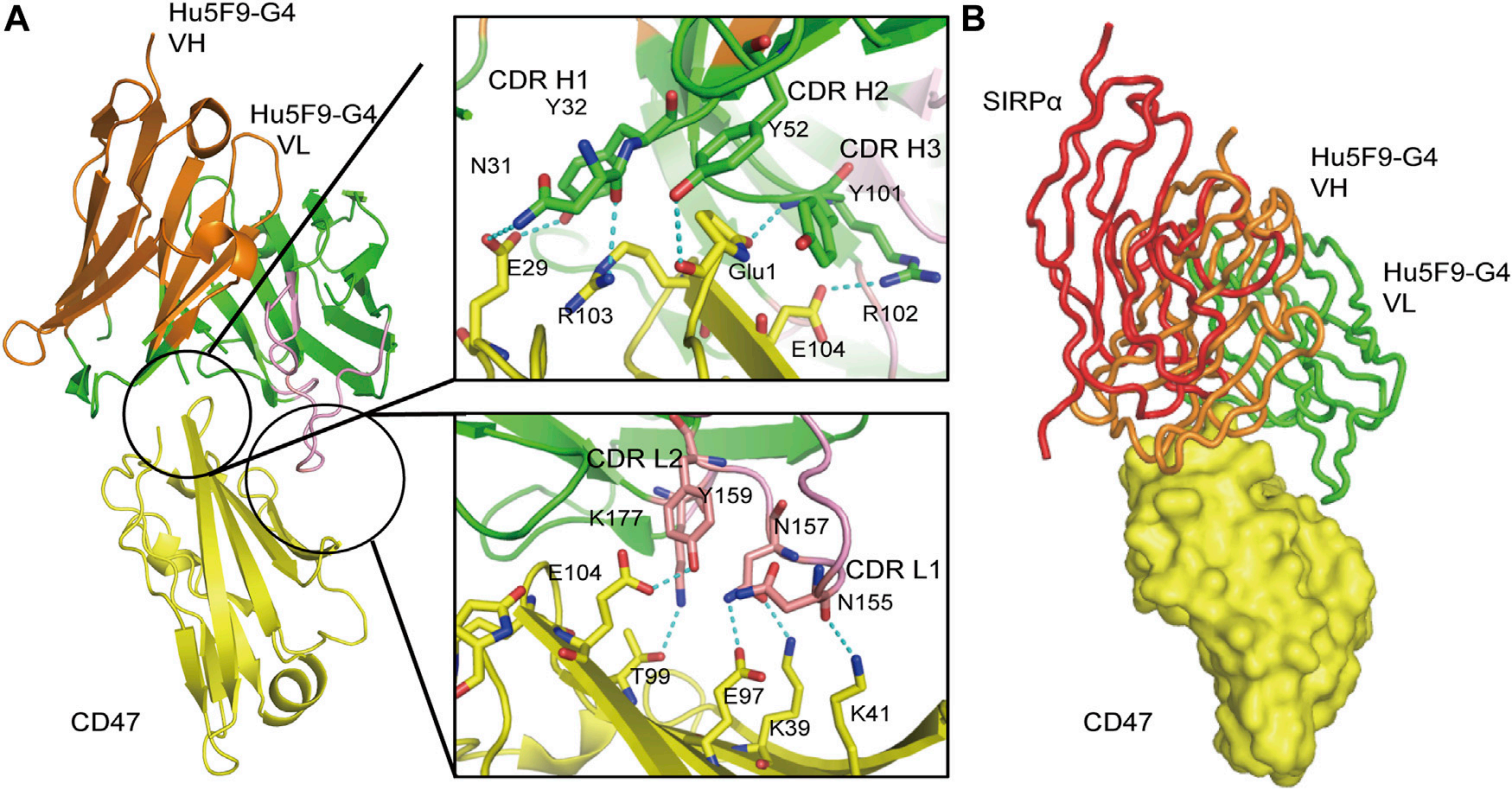
Three-dimensional (3D) structure diagram of the complex of Hu5F9-G4 and CD47 showed that Hu5F9-G4 had competitive antagonism with SIRPα. The orange and green peptide chains are the heavy chain and light chain of Hu5F9-G4, respectively. The light chain variable region peptide is represented in pink. The yellow peptide chain represents CD47, while the red peptide chain represents SIRPα. **(A)** Three-dimensional structure of CD47-ECD and Hu5F9-G4 complex after crystallization (PDB 5IWL). All complementary determiners of Hu5F9-G4 heavy chain and light chain variable CDR1 and CDR2 rings help Hu5F9-G4 bind CD47-ECD epitopes. **(B)** Hu5F9-G4 and SIRPα share binding pockets, which can be observed by superposition of Hu5F9-G4/CD47-ECD and SIRPα/CD47-ECD structures (PDB ID 2JJS).

Hu5F9-G4 (Magrolimab) is a monoclonal antibody that has been modified through humanization to target CD47. By competing with CD47, it inhibits CD47-induced macrophage phagocytosis and enhances antibody-dependent phagocytosis of macrophages to eliminate tumor cells (Figure 7) (Kim et al., 2008). Hu5F9-G4 is a human IgG4 antibody, which does not have antibody-dependent cell-mediated phagocytosis (ADCP) activity itself, but only carries the activity of blocking CD47-SIRPα interaction. Therefore, it must be combined with other antibody drugs with ADCC/ADCP activity to have good efficacy. Hu5F9-G4 can be used as a single drug, combined with chemotherapy drugs, combined with tumor targeting drugs and combined with immunotherapy. Currently, Gilead is in phase III development of Hu5F9-G4 and is working with azacitidine to treat MDS(NCT04313881) and acute myeloid leukemia (NCT04778397). Although the antibody was evaluated in phase III trials as a third-line treatment for metastatic triple-negative breast cancer, the study was stopped early due to lack of efficacy. Furthermore, phase II trials of Hu5F9-G4 are also underway for metastatic triple-negative breast cancer (NCT04958785) and head and neck squamous cell carcinoma (HNSCC) (NCT04854499). However, in clinical trials, Hu5F9-G4 has been associated with some high-risk adverse reactions, including anemia, neutropenia, and thrombocytopenia, most of which are grade 3 or 4. Thus, reducing the incidence of adverse events is the greatest challenge that Hu5F9-G4 must address. Despite this challenge, after implementing a low-dose priming dosing strategy (1 mg/kg in the first week and then a normal dose of 30 mg/kg in the second week), Hu5F9-G4 is already considered a promising immunological candidate for malignant tumors, particularly MDS and AML.

Evorpacept is a high-affinity CD47-blocking fusion protein with an inactive IgG Fc region and inhibits the binding of SIRPα to CD47. Therefore, Evorpacept can activate macrophage without harming red blood cells, which enables evorpacept to guarantee ideal efficacy in the treatment of solid tumors with less toxic side effects (Lakhani et al., 2021). It was developed by AXL Oncology for the treatment of various cancer therapies. Currently, Evorpacept is in phase II clinical trials for the treatment of metastatic/unresectable recurrent squamous cell carcinoma of the head and neck, with or without pembrolizumab (NCT04675333, NCT04675294, NCT05467670, etc.) (Lakhani et al., 2021). In 2020, the product received fast-track designation in the United States for the treatment of head and neck squamous cell carcinoma, as well as gastric or gastroesophageal cancer.

Lemzoparlimab is a whole-human monoclonal antibody against CD47 and is used as a single agent to treat relapsed or refractory acute myeloid leukemia (NCT04202003) and MDS in phase I/II (NCT04895410). Lemzoparlimab, administered weekly without any preexcitation, was well tolerated in the range from the lowest dose (1 mg/kg) to the highest dose (30 mg/kg), and no dose-limiting toxicity and hemolytic anemia were observed in all dose groups. At medium to high doses, the pharmacokinetic profile of single doses of lemzoparlimab was linear, with no significant antigenic “sinking effect” and no dose-limiting toxicity (DLT) events. Compared with conventional CD47 antibodies, the use of Lemzoparlimab effectively targets tumor cells while minimizing the adverse erythrocyte effects, thereby avoiding severe anemia (Junyuan et al., 2020).

Hu5F9-G4 is an earlier-developed CD47-targeting mAb. Since CD47 is also expressed by normal cells, Hu5F9-G4 also binds with normal cells expressing CD47, leading to adverse effects. To address this problem, researchers have developed anti-CD47-SIRPα axis fusion proteins such as Evorpacept, as well as enhanced targeting antibodies such as Lemzoparlimab, which minimizes binding to CD47 and reduces the occurrence of adverse reactions.

### 3.3 Bioactive peptides and small molecule inhibitors related to CD47-SIRPα signaling pathway

**FIGURE 8:**
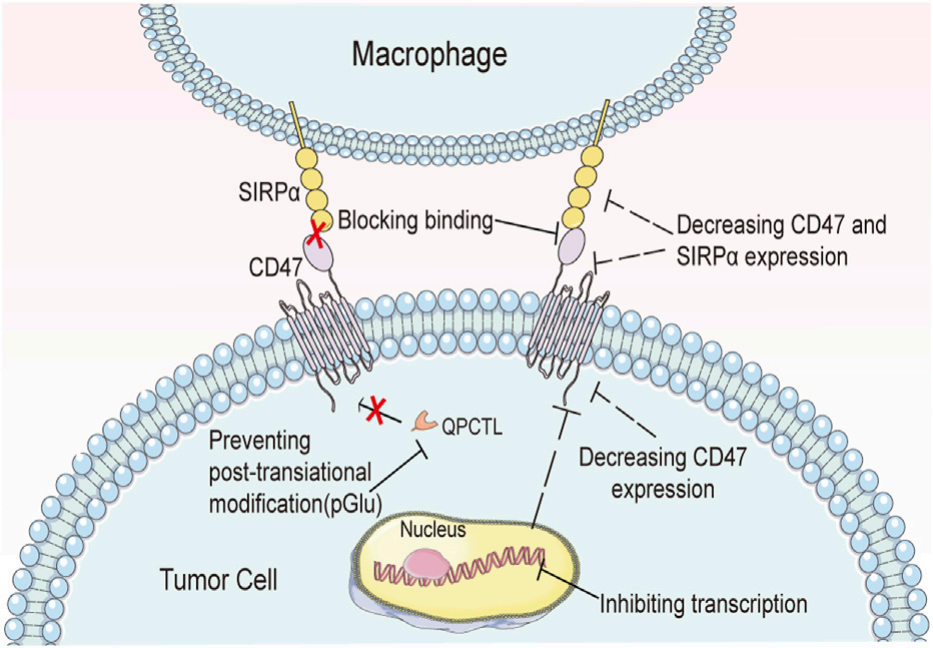
Biopeptides and small-molecule inhibitors affect signal transduction in the CD47-SIRPα axis in a variety of ways.

The mode of action of bioactive peptides and small molecule inhibitors against CD47-SIRPα axis is different from that of the above-mentioned antibodies and fusion proteins. Bioactive peptides cannot induce immune activation like antibodies, but instead, they only block the CD47-SIRPα signaling pathway. Meanwhile, small molecule inhibitors can enter cells and affect gene expression due to their small molecular weight. So far, they are divided into two classes according to two different mechanisms of action. The first class involves direct binding to CD47, thus blocking the CD47-SIRPα signal. Examples of this class include RS-17 and the 4-oxadiazole compound. The second class involves downregulating the expression of CD47-SIRPα signaling proteins. This is achieved by affecting transcription, translation, or post-translational modifications, thereby removing the immunosuppressive effects of the macrophages in this pathway. Examples of this class include RRX-001, PQ912, etc. (Figure 8).

#### 3.3.1 Bioactive peptide inhibitors

Bioactive peptide inhibitors block the signaling of CD47-SIRPα axis through their binding to CD47 or SIRPα. In order to achieve optimal anti-tumor effects, these inhibitors need to have a high affinity with their targets. Among them, Pep-20 and its derivatives, RS-17, D4-2, and SP5, have been extensively studied.

Pep-20 and its derivatives.

Wang et al. (2020) identified Pep-20 and its derivatives that interact with the CD47-SIRPα axis. Pep-20 is a straight peptide consisting of 12 amino acids that exhibits similar binding affinity toward human and mouse CD47, with Kd values of 2.91 ± 1.04 μM and 3.63 ± 1.71 μM, respectively. *In vitro* studies indicated that Pep-20 significantly promotes the recognition and killing function of macrophages in solid tumors and hematoma, while enhancing the ability of macrophages to mobilize T cells. *In vivo* experiments showed that Pep-20 inhibited tumor growth in mouse models. Importantly, unlike general anti-CD47-SIRPα axis drugs that can cause side effects such as erythrocytopenia, Pep-20 administered at a daily dose of 2 mg/kg did not produce significant erythrocyte reduction. Pep-20/CD47 docking models and alanine replacement experiments revealed that CD47 has three important sites that interact with Pep-20 and related inhibitors, located at Phe4, Glu104, and Glu106, respectively. Pep-20-D12, a more stable peptide variant with three D-amino acid residues, demonstrated superior anti-tumor efficacy compared to Pep-20 in colon tumor models (MC38). Additionally, the combination of Pep-20-D12 and interventional radiotherapy (IR) resulted in better elimination of existing tumors in mice than Pep-20-D12 alone.

RS—17.

Xu and Wang (2019) discovered RS-17 last year. RS-17 is a straight peptide consisting of 17 amino acids. RS-17 can effectively bind to human CD47, and the Kd value of both is 3.85 ± 0.79 nM. When RS-17 concentration is 20 μg/mL *in vitro*, the binding rate of human liver tumor cells (HepG2) to RS-17 is 71.2%, while that of epidermal squamous tumor cells (SCC-13) to RS-17 is 55.5%. In addition, *in vivo* experiments showed that RS-17 had significant inhibitory effect on tumor growth in mice which inoculated with hepatocellular carcinoma cells, and there was little effect on body weight and tumor volume in mice compared with control B6H12.

D4-2.

Hazama et al. (2020) has developed a series of anti-SIRPα cyclic peptides, among which D4-1, D4-2 and D4-4 have significant effects. These peptides bind to the extracellular domain of mouse SIRPα and block the recognition of SIRPα and CD47 through allosteric blocking. These peptides significantly improve the phagocytosis ability of macrophages, and inhibit the formation or metastasis of tumors *in vivo* with other antibodies such as CD20 or GP75 antibodies. No significant difference was found in Kd values of SIRPα and D4-2 in C57BL/6 and NOD mice. By analyzing the three-dimensional structure of the two complexes, the researchers found that the size of the interaction region of D4-2 on NOD SIRPα is 976.5Å^2^, and the two interact to form hydrogen bonds, hydrophobic bonds and salt Bridges. Ten amino acids except Tyr1, Tyr3, Tyr7, Ser8 and Cys14 in D4-2 participate in the formation of hydrogen bonds in the IgV domain of NOD SIRPα. The IgV-like structure of NOD SIRPα includes Asp84, which is easy to form electrostatic complex with Arg, and the second amino acid of D4-2 is Arg. A hydrophobic zone exists between D4-2 hydrophobic residues Ala5 and Pro11 and IgV-NOD SIRPα hydrophobic residues Phe51 and Phe56. Recognition of CD47 and SIRPα requires the participation of Phe56 and Ala65 in the C’E ring of NOD SIRPα IgV-like structure, so Phe56 and Ala65 are in a very critical position in signal transduction. When D4-2 binds to SIRPα, it changes its conformation and prevents CD47 from interacting with SIRPα.

SP5.

The Gao team at Zhengzhou University (Gao et al., 2020) designed a class of macrocyclic peptides containing SP1 to SP6 that can interfere with signal transduction by binding to SIRPα. Among them, SP4 and SP5 exhibited good affinity with SIRPα, with Kd values of 0.85 μM and 0.38 μM, respectively. As the dose increased, SP4 and SP5 exhibited enhanced blocking effects on the CD47-SIRPα axis. At a concentration of 200 μM, SP5 effectively promoted phagocytosis of human colon cancer cells (HT29) *in vitro*. In mouse colon cancer models and a mouse melanoma model (B16-OVA), SP5 demonstrated significant efficacy.

#### 3.3.2 Small molecule inhibitors

Compared with antibodies and other biological macromolecules and peptides, the advantages of existing small-molecule inhibitors lie in their small molecular weight and easy entry into tumor tissue. They can be broadly classified into two categories based on their mechanisms of action: the first type does not enter the cell and instead directly affects the binding of CD47 and SIRPα, while the second type enters the cell and reduces the production of proteins associated with CD47-SIRPα signaling by affecting transcription, translation, or post-translational modification. (See Table 1 for a summary.).

**TABLE 1:** Some small molecule inhibitors that affect transcription, translation, or posttranslational modifications to downregulate CD47-SIRPα signaling. RRx-001, metformin, 4-methylumbel ketone (4-Mu), JQ1, gefitinib and statins reduced CD47 expression by affecting transcription or translation processes. QPCTL regulates the formation of CD47 pyroglutamate, thereby facilitating its binding to SIRPα. At present, inhibitors that regulate QPCTL include SEN177 and PQ912 has entered clinical trials as a candidate for Alzheimer's disease. It has been proved that QPCTL inhibitors can block the recognition effect of CD47 on SIRPα and restore phagocytic function of macrophages, and enhance the anti-tumor effect *in vivo*.

Compound name	Combined structural formula	Action mechanism	Indication
RRx-001	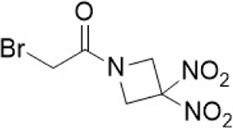	Downregulated protein expression	Small cell lung cancer
Metformin	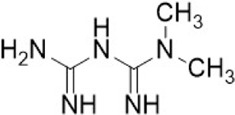	Downregulated protein expression	Breast cancer
4Mu	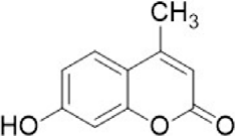	Downregulated protein expression	HCC
JQ1	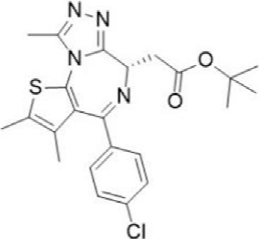	Downregulated protein expression	Lymphoma
Gefitinib	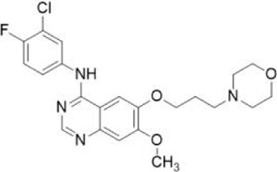	Downregulated protein expression	NSCLC
Statins	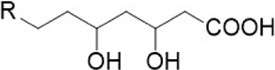	Downregulated protein expression	Atherosclerosis
PQ912	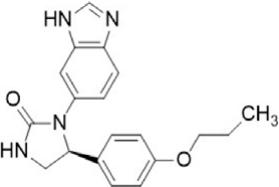	Affect protein post-translational modification	Melanoma
SEN177	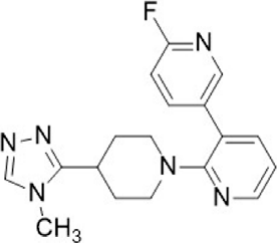	Affect protein post-translational modification	Melanoma

##### 3.3.2.1 Small molecule inhibitors that directly affect CD47 and SIRPα binding

NCGC00138783 and its derivatives.

**FIGURE 9:**
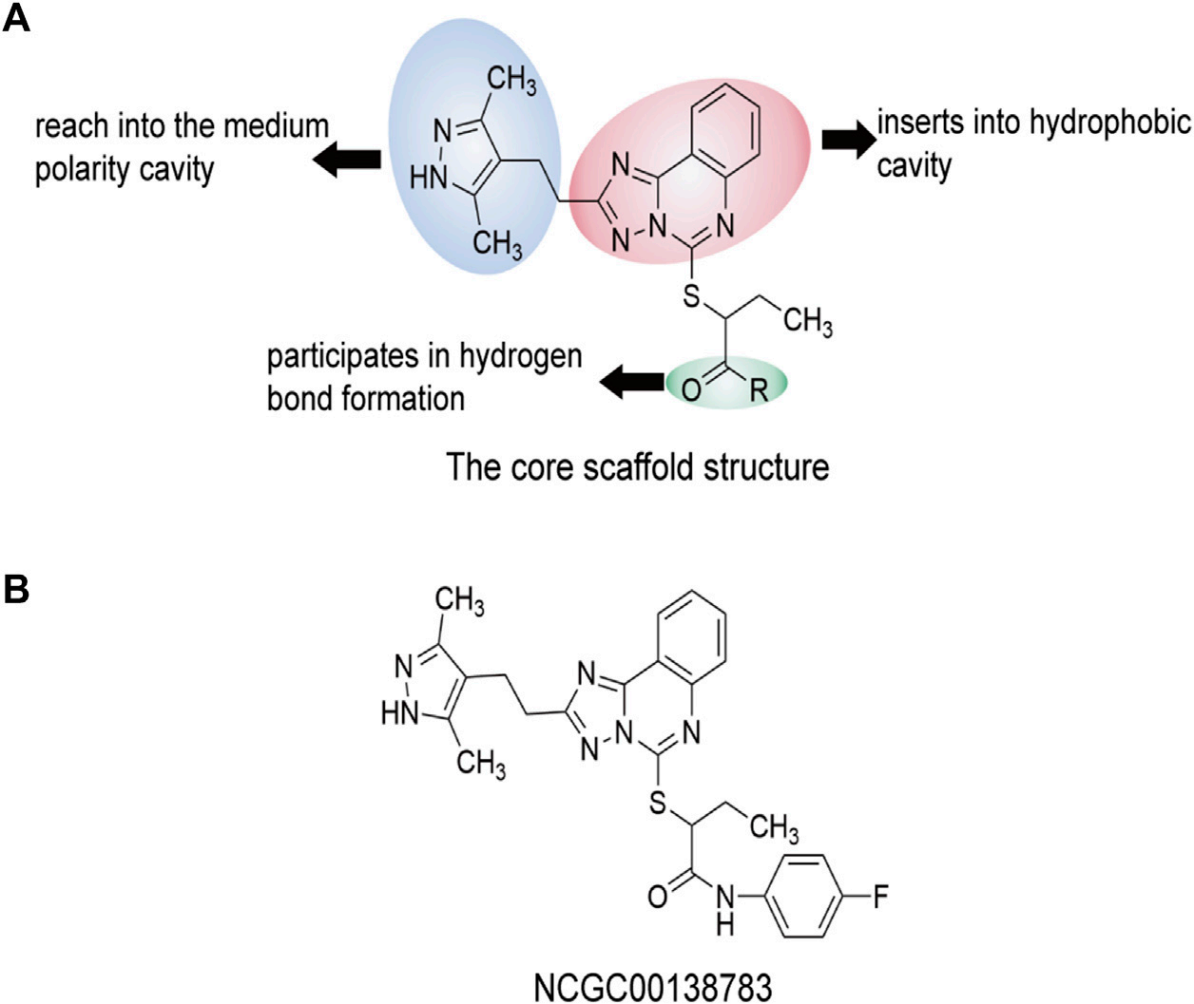
The core scaffold structure and chemical structure formula of NCGC00138783 (1). Predicted interaction regions between NCGC00138783 (1) and SIRPα are shown in yellow, the interaction regions of NCGC00138783 (1) are shown in cyan, and the related amino acids of SIRPα are shown in red. Compounds is the skeleton structure of 2—((2—(3, 5—dimethyl—1 h—pyrazole—4—alkali) ethyl)—5, 6—dihydro—[1, 4—trichlorobenzene] triazole [1, 5—c] quinazoline—5—alkali) sulfur butanol. **(A)** The core scaffold structure of NCGC00138783 (1). **(B)** The chemical structure formula of NCGC00138783 (1).

In recent years, researchers (Miller et al., 2019) have discovered the parent compound NCGC00138783 (1), which selectively binds to SIRPα and blocks the recognition of CD47. The skeletal structure of 2—((2 - (3, 5—dimethyl—1 h—pyrazole—4—base) ethyl)—5, 6—dihydro—[1, 4-trichlorobenzene] triazole [1, 5—c] quinazoline—5—base) sulfur butanol was later determined (Huang et al., 2021), with the IC_50_ of NCG00138783 (1) being 40 μM. The pharmacodynamic properties of NCGC00138783 (1) were further optimized to search for derivatives with similar functions. By combining acyl groups of NCGC00138783 (1) with monocyclic substituted amino and hydroxyl groups, a series of compounds with a high affinity for SIRPα can be obtained. In addition to the parent compound NCGC00138783 (1), NCG00538430 (2) and NCG00538419 (3) also inhibited the recognition between CD47 and SIRPα in alpha screening and LSC detection. In the docking experiment between NCGC00138783 (1) and CD47/SIRPα (PDB ID: 2JJT), NCGC00138783 (1) was more likely to bind to SIRPα than CD47. NCGC00138783 (1) interacts with three parts of SIRPα: the 3, 5-dimethyl-1-pyrazole group and the amide group located in the branch chain, and the (ElliottKosterMurphy, 2017; Yunna et al., 2020; KlocKubiak, 2021) triazoline [1, 5-C] quinazoline group located in the center. NCGC00138783 (1) and its derivatives form hydrogen bonds or T-superpositions with SIRPα sequence Leu30, Gly34, Pro35, Gln52, Lys53, and Lys93e. Where Gln52 is located, the polarity is large, which makes the polar groups of NCGC00138783 (1), such as amide groups, easy to produce hydrogen bond with it. These six amino acid sites participate in the recognition of SIRPα and CD47. Overall, NCGC00138783 (1) can bind SIRPα and occupy a key binding position in the CD47/SIRPα interaction (Figure 9).

1,2, 4-oxadiazole compound.

**FIGURE 10:**
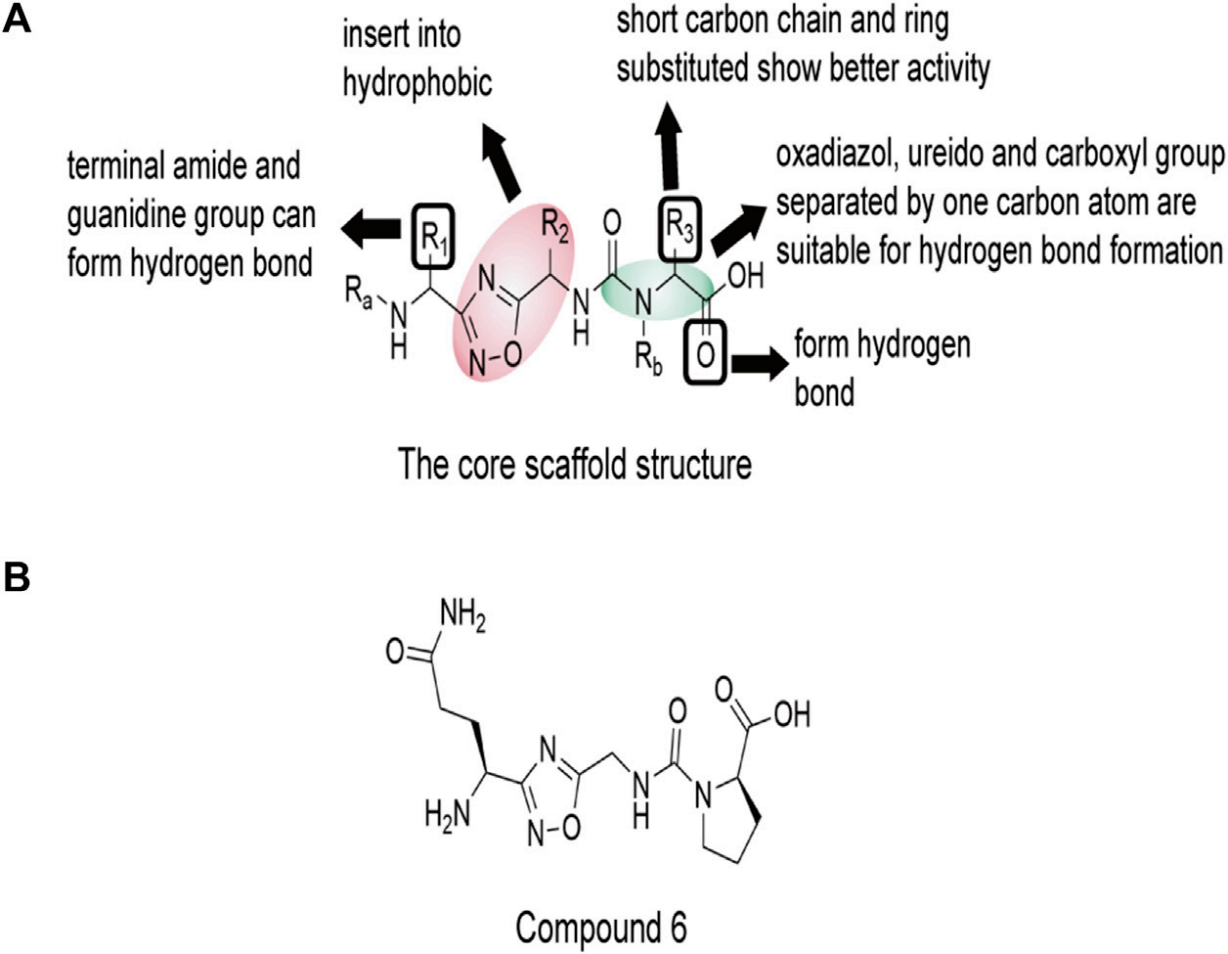
The core scaffold structure and chemical structure formula of compound 6 (1). The core 1,2, 4-diazole and butanamide groups would interact with Lys6, Trp40 and Thr107 in a hydrophobic region of CD47, which are also key sites for the recognition of CD47 and SIRPα. Thr7 and Thr107 near the core may form hydrogen bonds with the carbonyl group of butyamide. Asn5 is close to Lys6 of CD47, and may interact with two carbonyl groups of carbamoylproline. **(A)** The core scaffold structure of compound 6 (1). **(B)** The chemical structure formula of compound 6 (1).

Recently, Sasikumar’s group reported a group of small molecules that block CD47-SIRPα axis signal transduction using oxadiazole as the scaffold (Pottayil, 2019). These compounds increase macrophages’ ability to phagocytose human lymphoma and myeloma cells. In a mouse model of A20 with three different doses, the higher the dose of compound 6 (1), the better the tumor growth inhibition effect. In the predicted combination results of compound 6 (1) with CD47(PDB ID: 2JJT), the groups of 1,2, 4-diazole and butyamide have certain hydrophobicity, and can form hydrophobic regions with Lys6, Trp40 and Thr107 in CD47, which also have hydrophobic groups. These three amino acid sites are also the key sites for the recognition of CD47 and SIRPα. The carbonyl group of butyamide in compound 6 (1) forms hydrogen bonds with Thr7 and Thr107 of CD47. Asn5 may interact with two carbonyl groups of carbamoylproline because of its proximity to Lys6 of CD47 (Figure 10).

##### 3.3.2.2 Small-molecule inhibitors affecting transcription, translation, or posttranslational modifications downregulating CD47-SIRPα signaling expression

RRx—001.

RRx-001 was initially discovered in the aerospace industry and subsequently developed into an entirely new drug by EpicentRx. This immunomodulatory drug boasts an excellent safety profile and can reduce CD47 and SIRPα expression through transcription and translation levels, and promotes the transformation of TAM and other immune cells into immunostimulatory phenotypes. Additionally, it can improve blood flow distribution to tumor tissue and facilitate drug and oxygen delivery (Oronsky et al., 2017). As an emerging immunotherapy, RRx-001 can eliminate treatment resistance for patients and increase the sensitivity of tumor cells to drugs. Clinically, it can be used alone or in combination with radiotherapy and chemotherapy. RRx-001 is in phase III trial called REPLATINUM (NCT03699956), targeting third-line and higher small cell lung cancer (Oronsky et al., 2019). The metabolic characteristics of RRx-001 are that it preferentially binds to hemoglobin and displaces nitric oxide from the hemoglobin binding site, while nitric oxide can cause pain through pain receptors on the tissues around blood vessels and veins in the human body. The researchers mitigated the adverse effects of RRx-001 by mixing it with autologous blood *in vitro* (Caroen et al., 2020).

Metformin

Treatment with metformin has been shown to reduce the number of macrophages that differentiate into M2-type cells, leading to a higher proportion of M1-type macrophages compared to M2-type ones (Viollet et al., 2012; Tan et al., 2019; Yu et al., 2021). Metformin has also been identified as a CD47 inhibitor *in vitro* (Weiskopf et al., 2016), with breast cancer cells treated with 10 mM metformin exhibiting a 50% decrease in CD47 mRNA levels. However, the exact mechanism by which metformin regulates CD47 expression remains unclear.

4 Mu.

4 Mu has been shown to inhibit the normal synthesis of hyaluronic acid as well as reduce the production of mRNA and protein associated with CD47 in cells. Specifically, treating Hepa 129 cells with a concentration of 0.5 mM 4 Mu for 72 h led to a reduction in CD47 expression (Rodríguez et al., 2018). Moreover, CD47 mRNA levels in HCC mouse models were significantly decreased following 4Mu treatment (Rodríguez et al., 2021).

JQ1.

The bromodomain and extraterrestrial (BET) family proteins participate in the histone acetylation process, and JQ1 regulates histone acetylation by inhibiting BET (Da Motta et al., 2017). Various tumor cells have shown decreased production of CD47 protein after treatment with JQ1 through the c-Myc pathway. Specifically, JQ1 treatment at a concentration of 10 mM can suppress c-Myc and significantly decrease CD47 production at both transcriptional and translational levels (Wang et al., 2017). In addition, JQ1 (1μM) decreased the protein production of CD47 in MCF7 cells by inhibiting the binding of BRD4 to SEs (Betancur et al., 2017).

Gefitinib

Gefitinib has demonstrated superior efficacy in treating lung cancer due to its reversible binding to the ATP-binding domain of EGFR, inhibition of intracellular phosphorylation, and blocking of downstream signaling pathways (Sim et al., 2018). Recent studies have shown that the use of gefitinib can significantly reduce the expression of CD47 protein and increase the phagocytic capacity of DC cells (Kim et al., 2008). Specifically, treatment with gefitinib at a concentration of 50 nM led to a significant reduction in CD47 protein levels in PC9 cells (lung cancer cell) after 48 h. Combination therapy with a CD47 antibody further enhanced the phagocytic capacity of mouse macrophages compared to monotherapy (Nigro et al., 2020).

Statins.

Leeper and his team discovered that statins have a negative regulatory effect on the most active upstream signal of the CD47-SIRPα signal axis in apoptotic cells. When the researchers treated mice with atherosclerosis (high-fat diet, ApoE^−/−^), they found that the combination therapy using statins and a CD47-SIRPα inhibitor significantly reduced lesion size compared to statins alone or the inhibitor alone, and also decreased the necrotic core area. Mechanistically, the proinflammatory factor NF-κB1 in cells is a key transcription factor encoding the cd47 gene (CD47), which enters the nucleus and activates cd47 transcription. Statins can inhibit the nuclear transfer of NF-κB1, thereby inhibiting the expression of CD47 gene by apoptotic cells in atherosclerotic plaques. This directly disrupts the CD47-SIRPα signal axis of apoptotic cells and promotes macrophage phagocytosis and removal of accumulated apoptotic cells in atherosclerotic plaques (Jarr et al., 2022).

QPCTL inhibitors.

QPCT has been identified as an important regulator of the CD47-SIRPα signaling pathway. Specifically, QPCTL regulates the formation of CD47 pyroglutamic acid (pGlu), which interferes with its interaction with SIRPα. Interference with QPCTL activity has been shown to enhance the antibody-dependent phagocytic and cytotoxic capabilities of macrophages, while also improving the killing ability of neutrophils against tumor cells *in vivo* (Logtenberg et al., 2019). Inhibition of QPCTL function using SEN177 (Pozzi et al., 2018) and PQ912 (Scheltens et al., 2018) has been demonstrated to induce conformational changes in CD47. Furthermore, combination treatment with SEN177 and anti-CD20 antibody not only inhibits QPCTL function but also enhances anti-CD20 antibody-dependent phagocytosis (Logtenberg et al., 2019).

The two types of small molecule drugs that directly affect CD47 and SIRPα binding have unique structure-activity relationships, while the small molecule drugs that downregulate CD47-SIRPα signal expression by affecting transcription, translation or post-translational modification can regulate gene expression levels, but have no obvious structural correlation. In view of these characteristics, it is necessary to design inhibitors that block the recognition of CD47 and SIRPα by summarizing the structural characteristics, and optimize the structure of the derivatives of small molecule inhibitors that affect gene expression, which may further improve the targeting and safety of small molecule drugs.

CD47-SIRPα signaling pathway is the first “do not eat me” signaling pathway discovered by researchers. Currently, many forms of biomacromolecule and small molecule drugs have been developed and used in this pathway. Studies on CD47-SIRPα signaling pathway drugs can be divided into three levels: 1) AML/MDS is currently a relatively more confirmed indication for CD47-targeted drugs. 2) NHL and other hematoma are the exploration direction of CD47. Currently, preliminary efficacy data have been obtained, and the potential of combined therapy in the future needs further data verification. 3) Solid tumor is the ultimate target of CD47. Currently, multiple drug single-drug or combination strategies have shown efficacy in solid tumor. It is inevitable to find some adverse reactions caused by blocking this pathway and the occurrence of insensitivity and tolerance of some tumor cells. In the study of immunotherapy based on targeting macrophages, in addition to optimizing drug structure and enhancing drug targeting, researchers also need to find other therapeutic methods to cooperate with or make up for the deficiency of blocking CD47-SIRPα.

## 4 CD24-Siglec10 related drug development

In 2019, Weissman and his team discovered that CD24 was upregulated in several types of tumors. It is interesting to note that CD24 signaling often operates complementary to CD47 signaling. Some cancers, like blood cancers, tend to be highly sensitive to CD47 signaling blocking therapies but not to CD24 signaling inhibitors, while others, such as ovarian cancer, are more responsive to CD24 signaling blocking therapies. Notably, CD24 is most highly upregulated in tumor cells found in triple-negative breast cancer (TNBC) and ovarian cancer. Variations in CD24 expression levels can impact recurrence rates and survival in cancer patients. Those with low CD24 expression, both in ovarian and breast cancer, have significantly lower recurrence rates and an improved overall survival post-treatment (Jaiswal et al., 2009). CD24 has been shown to bind to Siglec10 molecules on macrophages, which in turn reduces macrophage phagocytosis and conveys a “do not eat me” signal (Bradley, 2019). This innate immune checkpoint, CD24-Siglec10, can be utilized to regulate macrophage-mediated anti-tumor immune responses in drug development.

Currently, the main focus of drug development in relation to the CD24-Siglec10 signaling pathway is centered around CD24. Regrettably, only two drugs, MK-7110 and exosome overexpressed CD24, have progressed to clinical stage testing. The preclinical work has focused on the development of anti-CD24 antibodies, including SWA-11, 12E9, cG7-sTGFBR2, cG7Q, and rG7S-MICA.

MK-7110 (CD24-Fc) is a recombinant fusion protein currently being evaluated in phase III clinical trials by Merck for the prevention of acute graft-versus-host disease (GVHD) (NCT04095858). The study involves two research groups. Patients in Group 1 receive intravenous CD24-Fc three times a month at a dose of 240 mg, doubled on day 1. Patients in Group 2 receive a 100 mL placebo (saline solution) infusion at the same time as Group 1, and tacrolimus is initiated on day 3 in both groups. Intravenous (0.03 mg/kg/day) or PO (0.045 mg/kg/dose) administration of tacrolimus is permitted. Methotrexate is administered intravenously at a dose of 15 mg/m2/dose on day 1 after HCT and 10 mg/m^2^/dose on days 3, 6, and 11 after HCT. Additionally, a phase II trial utilizing MK-7110 to reduce LDL and inflammation in HIV patients undergoing antiretroviral therapy is underway (NCT03960541) (Tian et al., 2018). MK-7110 is also being used in the clinical treatment of COVID-19, however, due to technical, clinical and regulatory uncertainties, as well as the fact that multiple drugs are currently available for the treatment of COVID-19 patients, and other more viable therapies and vaccines need to be invested, Merck has decided to abandon the follow-up study of MK-7110 for the treatment of COVID-19 (Welker et al., 2022; Eckhardt and O'Donnell, 2022; Song et al., 2022).

Exosomal overexpression of CD24 (EXO-CD24), administered *via* aerosol inhalation, is currently undergoing clinical phase II trials at Tel Aviv Sourasky Medical Center for the treatment of COVID-19 infection (NCT04969172). EXO-CD24 is an experimental drug with CD24 protein as a carrier. It delivers the drug CD24 into the body *via* vesicular exosomes, which regulate cytokine storms and thus resist COVID-19 (Shapira et al., 2022). EXO-CD24 has also been utilized in patients presenting moderate to severe cases of COVID-19 (Arber and Shapira, 2021; Krishnan et al., 2022).

SWA-11 is a monoclonal antibody that effectively inhibits the growth of lung and ovarian cancer xenografts by targeting CD24. The main effect of SWA-11 is to inhibit tumor cell proliferation. Following treatment with SWA-11, tumor vessel density significantly decreases, and the intratumoral cytokine environment is strongly impacted (Bretz N. et al., 2012; Baumann et al., 2012; Bretz NP. et al., 2012; Salnikov et al., 2013). Additionally, SWA-11 interferes with CD24 signaling and regulates the tumor microenvironment, which sensitizes tumor cells to gemcitabine chemotherapy. As such, the combination of anti-CD24 antibodies and chemotherapeutic drugs is more effective for cancer stem cells that are resistant to chemotherapy (Salnikov et al., 2013).

12E9, a high-affinity monoclonal antibody that binds to amino acids 27 to 59 of the CD24 surface receptor protein. It reduces the survival of many cancer cells, particularly breast cancer (SungurogluUgurAkcora Yildiz, 2021).

cG7-sTGFBR2 is a bispecific fusion protein consisting of the cG7 antibody against human CD24, in which the C-terminal heavy chain of the antibody is further fused to codon optimization. The extracellular region of human TGFB receptor 2 (TGFBR2) is specifically transformed by growth factor beta 1 (TGFB1) through a G4S linker. cG7-sTGFBR2 is able to transport TGFBR2 into the tumor microenvironment by targeting CD24. TGFBR2 can then inhibit TGF-β binding to TGF-β type II receptors on tumor cells by trapping free TGF-β (Zhang et al., 2019).

cG7Q was genetically engineered to mutate asparagine, a 297 conserved glycosylation site in CH2 region of a heavy-chain chimeric anti-human CD24 antibody, to glutamine. The mutant cG7Q retained the parental ability and could specifically bind to the surface of tumor cells. Compared with the parental antibody, the ADCC effect of the mutant cG7Q was slightly reduced, which helped to reduce the non-specific toxicity of the antibody and its conjugates. Among cG7Q molecules, glutamine is exposed at position 295, which can be catalyzed by glutamine transaminase to couple small toxic molecules to the modified mutant cG7Q, resulting in a variety of antigen-drug conjugates (ADCs). According to this strategy, four antibody-conjugated drugs have been prepared (Ma et al., 2016).

rG7S-MICA is also a bispecific fusion protein generated by fusing MHC-I-associated chain A (MICA) fraction with a scFv fragment that targets CD24. rG7S-MICA can bind to CD24 and NKG2D to enhance the sensitivity of NK cells to CD24 CRC cells and NKG2D-mediated immune surveillance. Additionally, rG7S-MICA enhances the killing of tumor cells by promoting NK cell recruitment and cytokine release in tumor tissues (Wang et al., 2018).

In existing studies on blocking the CD24-Siglec10 axis, anti-CD24 antibodies have been shown to effectively block the CD24-Siglec10 signaling pathway and activate the ability of macrophages to phagocytize tumor cells. Furthermore, numerous anti-CD24 mabs have been selected in combination with PD-1 for a significant anti-tumor effect. These findings suggest that CD24 is likely to be the next CD47, complementing related drugs targeting CD47 as “do not eat me” signaling molecules for tumor immunotherapy.

## 5 Conclusion and prospects

Both CD47-SIRPα axis and CD24-Siglec10 axis are hot research topics relating to the “do not eat me” signal. However, due to differences in when they were discovered, advancements in tumor immunotherapy using the two axes differ slightly. The CD24-Siglec10 signaling pathway was discovered later, and most drugs targeting it are in the preclinical stage. In contrast, drugs relating to the CD47-SIRPα axis, discovered earlier, have abundant clinical data. Presently, anti-CD47-SIRPα axis drugs that have entered the clinical trial phase demonstrate varying degrees of adverse reactions (Chao et al., 2020; Qu et al., 2022). For instance, in January 2022, the FDA temporarily suspended Gilead’s Magrolimab in clinical trials due to unexpected serious adverse effects (SUSAR). This adverse effect was related to erythrocyte/platelet toxicity created by the Magrolimab binding to CD47 on normal erythrocytes/platelets (Huang et al., 2020). CD24 and CD47 are both expressed abnormally on tumor cells and express in normal tissues (Oldenborg, 2013). However, in normal cells, only blocking the interaction of CD47-SIRPα is not enough to trigger phagocytosis, but also requires the assistance of “eat me” signals such as calreticulin to identify and engulf the cells. However, tumor cells, senescent red blood cells and platelets do not express “eat me” signals. Therefore, blocking the CD47-SIRPα interaction directly improves the macrophages’ ability to clear tumor cells, senescent red blood cells, and platelets, leading to adverse reactions (Khandelwal et al., 2007; Kwong et al., 2014).

Existing drugs targeting the “do not eat me” signaling pathway have effectively blocked the CD47-SIRPα axis or CD24-Siglec10 axis, enhancing the phagocytic ability of macrophages. These drugs have great potential in tumor immunotherapy, but several challenges still need to be addressed. Firstly, normal cells also express “do not eat me” signaling proteins, and drugs binding to these proteins may cause severe adverse reactions. Antibodies have high specificity, but they also have a long half-life in the body, leading to prolonged adverse reactions if they bind to CD47 or CD24 on normal cells. In contrast, small-molecule drugs have a lower risk of causing adverse events, but their lower affinity and selectivity mean that off-target effects may occur. Secondly, given the pervasive expression of “do not eat me” signaling proteins, drugs may need to be administered in high doses or frequently to achieve therapeutic blocking. Basic research has shown that drugs need to occupy 40%–60% of macrophage receptors to achieve the desired phagocytic effect. The clinical trial of Hu5F9-G4 used high therapeutic doses to achieve this goal, but the higher doses increased the risk of adverse reactions and reduced patient compliance. Moreover, selecting animal models for early drug evaluation is difficult. For example, the data from the SIRPα NOD mouse model showed that human CD47 had nearly 10 times higher binding affinity for murine SIRPα than for human SIRPα. Therefore, it is challenging to replicate these data in human clinical trials. Besides, SIRPα NOD mice lack critical mechanisms such as ADCC, which limits their use as a model for evaluating tumor immunotherapy. Finally, clinical drug development is a long and expensive process that may yield uncertain outcomes. Companies may face challenges in completing clinical trials for their drug candidates, and their development schedules may be affected by the clinical development schedules of their partners. Furthermore, the efficacy of CD47 products in solid tumors and other areas requires further validation, and their future progress remains highly uncertain.

Addressing the aforementioned challenges requires improving the tumor targeting of drugs and reducing their binding to “do not eat me” signaling proteins on normal cells, thereby decreasing their dosages and side effects. Several approaches can improve drug targeting. First, conditional activation drugs or prodrugs can be developed by searching for specific enzymes in tumors and designing drugs that are cleaved in the tumor microenvironment, exposing their binding epitopes and activating their binding activity. Nanoparticles and other drug delivery vectors targeting the tumor microenvironment can also enhance drug targeting. Second, antibody drugs can be designed by selecting appropriate IgG subtype antibody molecules through pre-exciting administration, screening, or designing unique antibody epitopes. For instance, reprogramming existing CD47 antibodies to the IgG4 subtype, instead of the IgG1 subtype, can reduce their adverse effects by weakening their ADCC and CDC effects. Moreover, finding new IgG4 subtype CD47 antibodies with better efficacy can be beneficial as this subtype has lower plasma content and limited toxic and side effects. Additionally, it is necessary to improve the target affinity of small molecule drugs to avoid the occurrence of off-target phenomenon. To achieve this, the eutectic structure of CD47-SIRPα or CD24-Siglec10 should be fully utilized to identify key binding sites, and drugs should be designed reasonably according to the structural characteristics of these sites to enhance their targeting. Moreover, to overcome the limitations of current animal models, gene editing technology could be used to select appropriate mice for modeling and monkeys or orangutans for early drug evaluation.

In conclusion, CD47, as a “do not eat me” signaling protein, has become a hot topic in drug research worldwide. CD47 and PD-1 target two major immune cell groups in tumor immunotherapy, with CD47 antibody mainly regulating macrophages and PD-1 antibody mainly regulating T lymphocytes. However, the development of CD47-SIRPα signaling pathway drugs is relatively slow, though they have enormous potential and could become comparable to PD-1/PD-L1 mAbs in the future. Meanwhile, CD24-Siglec10, as a congenital immune checkpoint, could also regulate macrophage-mediated anti-tumor immune responses for drug development. More data is needed to determine whether CD24 and CD47 work best complementarily. Nevertheless, we believe that this new strategy’s “do not eat me” message could save more lives in the future.

## Figures and Tables

**Table:** 

**ADCC**	antibody-dependent cytotoxic effects
**AML**	acute myelocytic leukemia
**ADCs**	antigen-drug conjugates
**ADCP**	antibody-dependent cell-mediated phagocytosis
**BET**	bromodomain and extraterrestrial
**CRT**	Cell surface calreticulin
**CDC**	complement-dependent cytotoxic effects
**DAMPs**	danger associated molecular patterns
**DLT**	dose-limiting toxicity
**EGF**	epidermal growth factor
**EV**	extracellular vesicles
**EXO-CD24**	Exosomal overexpression of CD24
**GVHD**	raft *versus* host disease
**HCC**	hepatocellular carcinoma
**HNSCC**	head and neck squamous cell carcinoma
**IAP**	integrin-associated protein
**ITIM**	immunoreceptor tyrosine inhibitory motif
**IR**	interventional radiotherapy
**LILRB1**	leukocyte immunoglobulin-like receptor B
**MHC-1**	major histocompatibility complex class I
**MDS**	myelodysplastic syndrome
**MICA**	major histocompatibility complex class I-associated chain A
**NK cells**	natural killer cells
**NHL**	non-Hodgkin’s lymphoma
**NSCLC**	non-small cell lung cancer
**RBCS**	red blood cells
**SCLC**	small cell lung cancer
**SIRPα**	signal regulatory protein α
**SHPS-1**	protein tyrosine phosphatase substrate 1
**SUSAR**	unexpected serious adverse effects
**SLAMF7**	Signaling lymphocyte activation molecular family 7
**TAM**	tumor-associated macrophages
**TNBC**	triple-negative breast cancer
**TGFBR2**	growth factor beta receptor 2
**TGFB1**	growth factor beta 1
**VEGF**	Vascular endothelial growth factor
**VAP-1**	vascular adhesion protein-1
