# Supplementary material for: New hope for tumor immunotherapy: the macrophage-related “do not eat me” signaling pathway
Source: Front Pharmacol. 2023 Jul 6;14:1228962. doi: 10.3389/fphar.2023.1228962 (PMC10358856; doi:10.3389/fphar.2023.1228962)
Supplement: Supplementary file 1 [file Table1.doc]

Supplementary Table S1. All drugs entering clinical trials by May 2023 on the CD47-SIRPα axis and CD24-Siglec10 axis are summarized in the table below. So far, most drugs in clinical studies target CD47 or SIRPα, while only four drugs or therapies targeting CD24 have been reported to have entered the clinical stage. Unfortunately, no drugs targeting Siglec10 have reached the clinical stage.

| Target | Drug Name | Phase | Mechanism of Action | Drug Characteristic | Indications | Organization | NTC number |
| --- | --- | --- | --- | --- | --- | --- | --- |
| CD47 | Lemzop-arlimab | Phase III | Anti-CD47 (Leukocyte Surface Antigen CD47), Drugs Targeting Tumor-Associated Macrophages Immune Checkpoint Inhibitors | Cancer Immunotherapy, Full Human Monoclonal Antibodies | Acute myeloid leukemia,  Solid tumor,  Lymphoma, Melanoma, Myelodysplasia | AbbVie I-Mab Biopharma (Originator) | NCT04895410 NCT04202003 NCT05709093 NCT05148533 NCT04912063 NCT03934814 |
|  | Magroli-mab | Phase III | Anti-CD47 (Leukocyte Surface Antigen CD47), Drugs Targeting Tumor-Associated Macrophages Immune Checkpoint Inhibitors | Cancer Immunotherapy, Humanized Monoclonal Antibodies | Acute myeloid leukemia,  urothelial carcinoma,  Brain cancer,  Breast (metastatic) cancer,  Colorectal cancer,  Head and neck (squamous cell carcinoma) cancer,  Ovary cancer,  Pancreas cancer,  Solid tumor, Fanconi anemia, Hematologic-blood cancer, Hodgkin lymphoma, (classical) Lymphoma,   1. cell Lymphoma,   Follicular Lymphoma,  Multiple myeloma, Myelodysplasia, Non-Hodgkin lymphoma, Triple negative breast cancer | Forty Seven (Gilead) Gilead Ono Stanford University (Originator) University of Oxford | NCT05330429  NCT04778410 NCT04778397 NCT04599634 NCT04435691 NCT02096770 NCT04827576 NCT04751383 NCT03558139  NCT02216409 NCT05738161 NCT05367401 NCT05079230 NCT04958785 NCT04892446 NCT04313881 NCT02678338 NCT04541017 NCT03922477 NCT03248479 NCT02953509 NCT05835011 NCT05829434 NCT02953782 |
|  | VT-1021 | Phase II/III | Drugs Targeting Thrombospondin-1 (THBS1; TSP1) | Peptides | Ovary cancer,  Pancreas cancer,  Solid tumor, Glioblastoma, Triple negative breast cancer | Vigeo (Originator) | NCT03364400 |
|  | Evorpa-cept | Phase II/III | Immune Checkpoint Inhibitors, Leukocyte Surface Antigen CD47/SIRPA Interaction Inhibitors, Phagocytosis Inducers | Cancer Immunotherapy, Fc Fusion Protein Consisting of 41 Amino Acids | Acute myeloid leukemia,  urothelial carcinoma,  Breast cancer,  Colorectal metastatic cancer,  Gastrointestinal cancer,  Ovary (epithelial) cancer,  Myelodysplasia, Non-Hodgkin lymphoma, | Alexo Therapeutics (ALX Oncology) ALX Oncology (Originator) | NCT04417517 NCT05787639 NCT05025800 NCT04755244 NCT05524545 NCT05167409 NCT04675294 NCT03013218 NCT05467670 NCT05027139 NCT05002127 NCT04675333 |
|  | HX-009 | Phase II | Anti-CD47 (Leukocyte Surface Antigen CD47), Anti-PDCD1 (Programmed Cell Death Protein 1; PD-1), Drugs Targeting Tumor-Associated Macrophages Immune Checkpoint Inhibitors | Bispecific Antibodies, Cancer Immunotherapy, Humanized Monoclonal Antibodies | Solid tumor | Waterstone Pharmaceuticals (Originator) | NCT04886271  NCT04097769 |
|  | Ligufali-mab | Phase II | Anti-CD47 (Leukocyte Surface Antigen CD47), Drugs Targeting Tumor-Associated Macrophages Immune Checkpoint Inhibitors | Cancer Immunotherapy, Humanized Monoclonal Antibodies | Acute myeloid leukemia,  Colorectal cancer,  Solid tumor, Myelodysplasia, Non-Hodgkin lymphoma, Triple negative breast cancer | Akeso Biopharma (Originator) | NCT04900350 NCT04728334 NCT04349969 NCT05382442 NCT05235542 NCT05227664  NCT04980885 |
|  | SIRPa-  IgG4 Fc | Phase II | Anti-CD47 (Leukocyte Surface Antigen CD47), Immune Checkpoint Inhibitors, Leukocyte Surface Antigen CD47/SIRPA Interaction Inhibitors, Phagocytosis Inducers | Cancer Immunotherapy, Fc Fusion Protein Consisting of 41 Amino Acids | Acute myeloid leukemia,  Lymphoma,  Diffuse large B-cell Multiple myeloma | Pfizer Trillium Therapeutics (Pfizer) University Health Network (UHN) (Originator) | NCT05261490 NCT05626322  NCT05567887 NCT03530683 NCT05507541 |
|  | SIRPa-  Fc | Phase II | Anti-CD47 (Leukocyte Surface Antigen CD47), Immune Checkpoint Inhibitors, Leukocyte Surface Antigen CD47/SIRPA Interaction Inhibitors, Phagocytosis Inducers | Fc Fusion Protein Consisting of 41 Amino Acids | Acute myeloid leukemia,  Solid tumor, T-cell lymphoma, Leiomyosarcoma, Diffuse large B-cell Mycosis fungoides, Peripheral T-cell lymphoma, Sezary syndrome | Pfizer Trillium Therapeutics (Pfizer) University Health Network (UHN) (Originator) | NCT02890368 NCT02663518 NCT05507541 NCT04996004 |
|  | 6MW-3211 | Phase I/II | Anti-CD274 (PD-L1), Anti-CD47 (Leukocyte Surface Antigen CD47), Drugs Targeting Tumor-Associated Macrophages Immune Checkpoint Inhibitors | Bispecific Antibodies, Cancer Immunotherapy | Acute myeloid leukemia,  Kidney (renal cell carcinoma, clear cell) cancer,  Lymphoma, Myelodysplasia | Mabwell (Shanghai) Bioscience (Originator) | NCT05048160  NCT05431569 NCT05448599 NCT05446688 |
|  | ISB-1442 | Phase I/II | Anti-CD38 (ADP-Ribosyl Cyclase/Cyclic ADP-Ribose Hydrolase 1), Anti-CD47 (Leukocyte Surface Antigen CD47), Drugs Targeting Tumor-Associated Macrophages Immune Checkpoint Inhibitors | Bispecific Antibodies, Cancer Immunotherapy | Multiple myeloma | Ichnos Sciences (Originator) | NCT05427812 |
|  | IMM-01 | Phase I/II | Drugs Targeting Leukocyte Surface Antigen CD47, Drugs Targeting Tumor-Associated Macrophages Immune Checkpoint Inhibitors | Cancer Immunotherapy, Fc Fusion Protein Consisting of 41 Amino Acids | Acute myeloid leukemia,  Solid tumor, Hodgkin lymphoma ,  Myelodysplasia, Myeloid leukemia | ImmuneOnco (Originator) | NCT05140811 |
|  | CPO-107 | Phase I/II | Drugs Targeting B-Lymphocyte Antigen CD20, Drugs Targeting Leukocyte Surface Antigen CD47 | Fc Fusion Protein Consisting of 41 Amino Acids | Lymphoma,  Non-Hodgkin lymphoma | China Shijiazhuang Pharmaceutical (CSPC) (CSPC Pharmaceutical Group) CSPC Pharmaceutical Group (Originator) | NCT04853329 |
|  | IMM-2902 | Phase I/II | Drugs Targeting HER2 (erbB2), Drugs Targeting Leukocyte Surface Antigen CD47, Signal Transduction Modulators | Cancer Immunotherapy, Fc Fusion Protein Consisting of 41 Amino Acids | Lung cancer,  Solid tumor, Cholangiocarcinoma | ImmuneOnco (Originator) | NCT05076591 NCT05805956 |
|  | IMM-0306 | Phase I/II | Anti-CD20, Drugs Targeting Leukocyte Surface Antigen CD47 | Cancer Immunotherapy, Fc Fusion Protein Consisting of 41 Amino Acids | B-cell Lymphoma | ImmuneOnco (Originator) | NCT05771883 |
|  | SIRPa-  41BB L | Phase I/II | Drugs Targeting Leukocyte Surface Antigen CD47, Tumor Necrosis Factor Receptor Superfamily Member 9 (TNFRSF9, CD137, 4-1BB) Ligands | Cancer Immunotherapy, Fc Fusion Protein Consisting of 41 Amino Acids | Acute myeloid leukemia,  NSCLC,  Solid tumor,  Myelodysplasia | KAHR Medical (Originator) | NCT04440735 |
|  | Letapli-mab | Phase I/II | Anti-CD47 (Leukocyte Surface Antigen CD47), Drugs Targeting Tumor-Associated Macrophages Immune Checkpoint Inhibitors, Signal Transduction Modulators | Cancer Immunotherapy, Full Human Monoclonal Antibodies | Acute myeloid leukemia, Ovary cancer,  Solid tumor, Myelodysplasia, Non-Hodgkin lymphoma | Innovent Biologics (Originator) | NCT04511975 NCT04485052  NCT03717103 NCT04485065 NCT03763149 |
|  | AO-176 | Phase I/II | Anti-CD47 (Leukocyte Surface Antigen CD47), Drugs Targeting Tumor-Associated Macrophages Immune Checkpoint Inhibitors | Cancer Immunotherapy, Humanized Monoclonal Antibodies | Solid tumor, Lymphoma, Multiple myeloma | Arch Oncology (Originator) | NCT04445701  NCT03834948 |
|  | BAT-7104 | Phase I | Anti-CD274 (PD-L1), Anti-CD47 (Leukocyte Surface Antigen CD47), Immune Checkpoint Inhibitors | Bispecific Antibodies, Cancer Immunotherapy | Solid tumor | Bio-Thera Solutions (Originator) | NCT05200013 NCT05767060 |
|  | QL-401 | Phase I | Anti-CD274 (PD-L1), Anti-CD47 (Leukocyte Surface Antigen CD47), Immune Checkpoint Inhibitors | Bispecific Antibodies, Cancer Immunotherapy, Full Human Monoclonal Antibodies | Cancer | QLSF Biotherapeutics (Originator) | NF |
|  | AUR-103 | Phase I | Immune Checkpoint Inhibitors, Leukocyte Surface Antigen CD47 Antagonists | 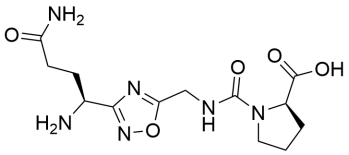 | Solid tumor | Aurigene (Originator) | NF |
|  | SG-2501 | Phase I | Anti-CD38 (ADP-Ribosyl Cyclase/Cyclic ADP-Ribose Hydrolase 1), Anti-CD47 (Leukocyte Surface Antigen CD47), Immune Checkpoint Inhibitors | Bispecific Antibodies, Cancer Immunotherapy | Lymphoma | Hangzhou Sumgen Biotechnology (Originator) | NCT05293912 |
|  | HMPL-A83 | Phase I | Anti-CD47 (Leukocyte Surface Antigen CD47), Drugs Targeting Tumor-Associated Macrophages Immune Checkpoint Inhibitors | Cancer Immunotherapy, Monoclonal Antibodies | Cancer | HUTCHMED (Originator) | NCT05429008 |
|  | SG-404 | Phase I | Immune Checkpoint Inhibitors, Leukocyte Surface Antigen CD47/SIRPA Interaction Inhibitors, Phagocytosis Inducers | Cancer Immunotherapy, Fc Fusion Protein Consisting of 41 Amino Acids | Acute myeloid leukemia,  Solid tumor, Lymphoma, Myelodysplasia | CNBG Sumgen Hangzhou Sumgen Biotechnology (Originator) | NF |
|  | PF-07257876 | Phase I | Anti-CD274 (PD-L1), Anti-CD47 (Leukocyte Surface Antigen CD47), Drugs Targeting Tumor-Associated Macrophages Immune Checkpoint Inhibitors | Bispecific Antibodies, Cancer Immunotherapy | Head and neck cancer,  NSCLC | Pfizer (Originator) | NCT04881045 |
|  | TQB-2928 | Phase I | Drugs Targeting Leukocyte Surface Antigen CD47 Immune Checkpoint Inhibitors, Leukocyte Surface Antigen CD47/SIRPA Interaction Inhibitors, Phagocytosis Inducers | NF | Solid tumor | Jiangsu Chia Tai Tianqing Pharmaceutical (Originator) | NCT04854681 NCT05192512 |
|  | STI-6643 | Phase I | Anti-CD47 (Leukocyte Surface Antigen CD47), Drugs Targeting Tumor-Associated Macrophages Immune Checkpoint Inhibitors | Cancer Immunotherapy, Full Human Monoclonal Antibodies | Solid tumor | Sorrento Therapeutics (Originator) | NCT04900519 |
|  | PT-886 | Phase I | Anti-CD47 (Leukocyte Surface Antigen CD47), Anti-CLDN18.2 (Claudin 18.2), Drugs Targeting Tumor-Associated Macrophages Immune Checkpoint Inhibitors | Bispecific Antibodies, Cancer Immunotherapy, Humanized Monoclonal Antibodies | Gastroesophageal junction cancer,  Pancreas cancer,  Stomach cancer | Phanes Therapeutics (Originator) | NCT05482893 |
|  | SG-12473 | Phase I | Anti-CD274 (PD-L1), Drugs Targeting Leukocyte Surface Antigen CD47 Immune Checkpoint Inhibitors | Bispecific Antibody Fusions, Cancer Immunotherapy, Humanized Monoclonal Antibodies, Fc Fusion Protein Consisting of 41 Amino Acids | Solid tumor | Hangzhou Sumgen Biotechnology (Originator) | NF |
|  | IBI-322 | Phase I | Anti-CD274 (PD-L1), Anti-CD47 (Leukocyte Surface Antigen CD47), Drugs Targeting Tumor-Associated Macrophages Immune Checkpoint Inhibitors | Bispecific Antibodies, Cancer Immunotherapy | Solid tumor | Innovent Biologics (Originator) | NCT04338659 NCT04912466 NCT04795128 NCT05148442 NCT04328831 |
|  | ZL-1201 | Phase I | Anti-CD47 (Leukocyte Surface Antigen CD47), Drugs Targeting Tumor-Associated Macrophages Immune Checkpoint Inhibitors | Antibodies, Cancer Immunotherapy | Solid tumor, Lymphoma | ZAI Lab (Originator) | NCT04257617 |
|  | Gentuli-zumab | Phase I | Anti-CD47 (Leukocyte Surface Antigen CD47), Drugs Targeting Tumor-Associated Macrophages Immune Checkpoint Inhibitors | Antibodies, Cancer Immunotherapy | Acute myeloid leukemia,  Solid tumor, Myelodysplasia, Non-Hodgkin lymphoma | GeneScience Pharmaceuticals (Originator) | NCT05221385 NCT05263271 |
|  | SIRPα- Fc-  CD40L | Phase I | Drugs Targeting CD40 Ligand (CD40LG; CD154), Drugs Targeting Leukocyte Surface Antigen CD47 | Fc Fusion Protein Consisting of 41 Amino Acids | Acute myeloid leukemia,  Fallopian tube cancer,  Head and neck (squamous cell carcinoma) Cancer,  Ovary Cancer,  Skin (squamous cell carcinoma) carcinoma, Primary peritoneal (ovarian), Myelodysplasia | Shattuck Labs (Originator) | NCT04406623 NCT05275439 NCT05483933 NCT04502888 |
|  | IMC-002 | Phase I | Anti-CD47 (Leukocyte Surface Antigen CD47), Drugs Targeting Tumor-Associated Macrophages Immune Checkpoint Inhibitors | Cancer Immunotherapy, Full Human Monoclonal Antibodies | solid tumor, Lymphoma | 3D Medicines ImmuneOncia Therapeutics Sorrento Therapeutics (Originator) | NCT05276310 NCT04306224 |
|  | AU7R-104 | Phase I | Fatty Acid Binding Protein, Epidermal (E-FABP; FABP5; KFABP) Inhibitors, Immune Checkpoint Inhibitors, Leukocyte Surface Antigen CD47 Antagonists, Leukocyte Surface Antigen CD47/SIRPA Interaction Inhibitors, Phagocytosis Inducers | Cancer Immunotherapy | Colorectal cancer, Chronic lymphocytic leukemia, Lymphoma,  Diffuse large B-cell (activated B-cell like) Lymphoma,  Non-Hodgkin lymphoma, Small lymphocytic lymphoma | Aurigene (Originator) Exelixis | NCT05144347 |
|  | SRF-231 | Phase I | Anti-CD47 (Leukocyte Surface Antigen CD47), Drugs Targeting Tumor-Associated Macrophages Immune Checkpoint Inhibitors | Cancer Immunotherapy, Full Human Monoclonal Antibodies | Solid tumor, Chronic lymphocytic Leukemia, Lymphoma,  Burkitt Multiple myeloma | Surface Oncology (Originator) | NCT03512340 |
|  | BI-765063 | Phase I | Anti-SIRPA , Drugs Targeting Tumor-Associated Macrophages Immune Checkpoint Inhibitors, Leukocyte Surface Antigen CD47/SIRPA Interaction Inhibitors, Phagocytosis Inducers | Cancer Immunotherapy, Humanized Monoclonal Antibodies | Colorectal cancer, Endometrium cancer,  Liver (hepatocellular carcinoma) cancer,  NSCLC  Solid tumor, Melanoma | Boehringer Ingelheim OSE Immunotherapeutics (Originator) | NCT03990233 NCT05249426 NCT05446129 NCT04653142 |
|  | CC-90002 | Phase I | Anti-CD47 (Leukocyte Surface Antigen CD47), Drugs Targeting Tumor-Associated Macrophages Immune Checkpoint Inhibitors | Cancer Immunotherapy, Monoclonal Antibodies | Acute myeloid leukemia, Myelodysplasia, Solid tumor, Non-Hodgkin lymphoma | Celgene Inhibrx (Originator) | NCT02367196 NCT02641002 |
|  | NI-1801 | Phase I | Anti-CD47 (Leukocyte Surface Antigen CD47), Anti-Mesothelin, Drugs Targeting Tumor-Associated Macrophages Immune Checkpoint Inhibitors | Bispecific Antibodies, Cancer Immunotherapy | NSCLC,  Ovary (epithelial) cancer,  Solid tumor, Triple negative breast cancer | Light Chain Bioscience (Originator) | NCT05403554 |
|  | NI-1701 | Phase I | Anti-CD19, Anti-CD47 (Leukocyte Surface Antigen CD47), Drugs Targeting Tumor-Associated Macrophages Immune Checkpoint Inhibitors | Bispecific Antibodies, Cancer Immunotherapy | Chronic lymphocytic leukemia, B-cell Lymphoma, | Light Chain Bioscience (Originator) NovImmune (Light Chain Bioscience) TG Therapeutics | NCT04806035 NCT03804996 |
| SIRPα | Evorpa-cept | Phase II/III | Immune Checkpoint Inhibitors, Leukocyte Surface Antigen CD47/SIRPA Interaction Inhibitors, Phagocytosis Inducers | Cancer Immunotherapy, Fc Fusion Protein Consisting of 41 Amino Acids | Acute myeloid leukemia,  Bladder (urothelial carcinoma) cancer,  Breast cancer,  Colorectal metastatic cancer,  Gastroesophageal Junction cancer,  Ovary (epithelial) cancer,  Solid tumor, Stomach cancer,  Myelodysplasia, Non-Hodgkin lymphoma | Alexo Therapeutics (ALX Oncology) ALX Oncology (Originator) | NCT04417517 NCT05787639 NCT05025800 NCT04755244 NCT05524545 NCT05167409 NCT04675294 NCT03013218 NCT05467670 NCT05027139 NCT05002127 NCT04675333 |
|  | LM-101 | Phase I/II | Anti-SIRPA | Cancer Immunotherapy, Monoclonal Antibodies | Recurrent or refractory advanced malignant tumors | LaNova Medicines (Originator) | NF |
|  | Anzurst-obart | Phase I | Anti-SIRPA , Drugs Targeting Tumor-Associated Macrophages | Cancer Immunotherapy, Monoclonal Antibodies | Solid tumor | Celgene (Originator) | NCT05168202 |
| CD24 | Neltepe-ndocel | Registered - 2023 | Human ex vivo expanded CD166+ CD24- CD105- CD44- CD26- CD200- allogeneic corneal endothelial cells derived from human corneal tissue | Cell Therapy, an allogeneic cell therapy with cultured corneal endothelial cells. | Corneal disorder Keratopathy | Aurion Biotech (Originator) | NF |
|  | MK-7110 | Phase III | Anti-CD24 (Signal Transducer CD24) | Fc Fusion Protein Consisting of 41 Amino Acids | Dyslipidemia Graft-versus-host disease Infection,  HIV, Multiple sclerosis, Rheumatoid arthritis, Severe acute respiratory syndrome coronavirus 2 (SARS-CoV-2) infection (COVID-19) | Merck & Co (Originator) Merck & Co. (Merck & Co) OncoImmune (Merck & Co) | NCT04317040 NCT04060407 NCT04095858 NCT03960541 NCT04552704 NCT02663622 |
|  | EXO-CD24 | Phase II | Anti-CD24 (Signal Transducer CD24) | Cell-Derived Exosomes, Polypeptides from 41 AA, Recombinant proteins | Severe acute respiratory syndrome coronavirus 2 (SARS-CoV-2) infection (COVID-19) | OBCT CD24 Tel Aviv Sourasky Medical Center (TASMC) | NCT04969172 NCT04902183 NCT04747574 |
|  | ATG-031 | Phase I | Anti-CD24 (Signal Transducer CD24) | Cancer Immunotherapy, Humanized Monoclonal Antibodies | Advanced solid tumors,  B-cell non-Hodgkin lymphoma (B-NHL) | Antengene | NF |
